# Nucleotide diversity maps reveal variation in diversity among wheat genomes and chromosomes

**DOI:** 10.1186/1471-2164-11-702

**Published:** 2010-12-14

**Authors:** Eduard D Akhunov, Alina R Akhunova, Olin D Anderson, James A Anderson, Nancy Blake, Michael T Clegg, Devin Coleman-Derr, Emily J Conley, Curt C Crossman, Karin R Deal, Jorge Dubcovsky, Bikram S Gill, Yong Q Gu, Jakub Hadam, Hwayoung Heo, Naxin Huo, Gerard R Lazo, Ming-Cheng Luo, Yaqin Q Ma, David E Matthews, Patrick E McGuire, Peter L Morrell, Calvin O Qualset, James Renfro, Dindo Tabanao, Luther E Talbert, Chao Tian, Donna M Toleno, Marilyn L Warburton, Frank M You, Wenjun Zhang, Jan Dvorak

**Affiliations:** 1 Department of Plant Sciences, University of California, Davis, CA 95616, USA; 2 Department of Plant Pathology, KSU, Manhattan, KS 66506, USA; 3 Genomics and Gene Discovery Unit, USDA/ARS Western Regional Research Center, Albany, CA 94710, USA; 4 Department of Agronomy and Plant Genetics, University of Minnesota, St. Paul, MN 55108, USA; 5 Department of Plant Sciences and Plant Pathology, Montana State University, Bozeman, MT 59717, USA; 6 Department of Ecology and Evolutionary Biology, University of California, Irvine, CA 92697, USA; 7 Department of Plant Pathology, Kansas State University, Manhattan KS 66506, USA; 8 Department of Botany and Plant Sciences, University of California, Riverside, CA 92521, USA; 9 USDA-ARS, Cornell University, Ithaca, NY 14853, USA; 10 Philippine Rice Research Institute, Maligaya, Nueva Ecija, Philippines; 11 The International Maize and Wheat Improvement Center (CIMMYT), 06600 Mexico, D.F., Mexico; 12 Corn Host Plant Research Resistance Unit, USDA/ARS MSU MS 39762, USA

## Abstract

**Background:**

A genome-wide assessment of nucleotide diversity in a polyploid species must minimize the inclusion of homoeologous sequences into diversity estimates and reliably allocate individual haplotypes into their respective genomes. The same requirements complicate the development and deployment of single nucleotide polymorphism (SNP) markers in polyploid species. We report here a strategy that satisfies these requirements and deploy it in the sequencing of genes in cultivated hexaploid wheat (*Triticum aestivum*, genomes AABBDD) and wild tetraploid wheat (*Triticum turgidum *ssp. *dicoccoides*, genomes AABB) from the putative site of wheat domestication in Turkey. Data are used to assess the distribution of diversity among and within wheat genomes and to develop a panel of SNP markers for polyploid wheat.

**Results:**

Nucleotide diversity was estimated in 2114 wheat genes and was similar between the A and B genomes and reduced in the D genome. Within a genome, diversity was diminished on some chromosomes. Low diversity was always accompanied by an excess of rare alleles. A total of 5,471 SNPs was discovered in 1791 wheat genes. Totals of 1,271, 1,218, and 2,203 SNPs were discovered in 488, 463, and 641 genes of wheat putative diploid ancestors, *T. urartu*, *Aegilops speltoides*, and *Ae. tauschii*, respectively. A public database containing genome-specific primers, SNPs, and other information was constructed. A total of 987 genes with nucleotide diversity estimated in one or more of the wheat genomes was placed on an *Ae. tauschii *genetic map, and the map was superimposed on wheat deletion-bin maps. The agreement between the maps was assessed.

**Conclusions:**

In a young polyploid, exemplified by *T. aestivum*, ancestral species are the primary source of genetic diversity. Low effective recombination due to self-pollination and a genetic mechanism precluding homoeologous chromosome pairing during polyploid meiosis can lead to the loss of diversity from large chromosomal regions. The net effect of these factors in *T. aestivum *is large variation in diversity among genomes and chromosomes, which impacts the development of SNP markers and their practical utility. Accumulation of new mutations in older polyploid species, such as wild emmer, results in increased diversity and its more uniform distribution across the genome.

## Background

While nucleotide diversity studies and the development and deployment of single nucleotide polymorphism (SNP) markers are straightforward in diploid and paleopolyploid species, such as maize or soybean [1-3], they are complicated in recently evolved polyploid species by high levels of orthologous gene similarity. Sequence similarity makes sequencing of single genes and allocation of sequences into respective genomes difficult. Special strategies are therefore required for nucleotide diversity studies and the development of SNP markers for young polyploid species, which include wheat and other economically important plants.

Wheat forms an allopolyploid series at three ploidy levels: diploid (2*x *= 14), tetraploid (4*x *= 28), and hexaploid (6*x *= 42). Wild tetraploid emmer wheat (*Triticum turgidum *ssp. *dicoccoides*, henceforth shortened to *T. dicoccoides*, genomes AABB) evolved between 0.2 and 0.5 million years ago [4,5] via hybridization of wild *T. urartu *(genomes AA) and an extinct or undiscovered species in the lineage of *Aegilops speltoides *(genomes SS, where S is closely related but not identical to the wheat B genome) [4,6-9]. Hexaploid wheat (*T. aestivum*, genomes AABBDD) evolved about 8,500 years ago [10] via hybridization of *T. turgidum *with diploid *Ae. tauschii *(genomes DD) [11,12].

A possible strategy for nucleotide diversity studies and SNP discovery in young polyploid species, such as wheat, is to find diverged regions in orthologous genes and use them for the design of polymerase chain reaction (PCR) primers that anneal to only a single DNA target. These genome-specific primers (GSPs) amplify DNA from only a single genome and facilitate gene sequencing and SNP discovery [13]. An alternative strategy is to shotgun-sequence cDNAs and then allocate each sequence to a genome. Both approaches have been used in polyploid wheat [13-15] although those studies were of limited scope and genome coverage [14-17] and none mapped the markers.

A domestication bottleneck at the tetraploid level and a polyploidy bottleneck during the transition from the tetraploid to hexaploid level are expected to have reduced the diversity of polyploid wheat compared to wild emmer. Nucleotide diversity *θ_π _*[18] was reported to be 2.7 × 10^-3 ^in 24 A- and B-genome wild emmer genes [16]. For comparison, *θ_π _*was estimated to be 9.7 × 10^-3 ^in teosinte genes (*Zea mays *ssp. *parviglumis*) [3] and 7.7 to 8.1 × 10^-3 ^in wild barley genes (*Hordeum vulgare *ssp. *spontaneum*) [19,20]. The diversity of emmer was reduced by the domestication bottleneck but, curiously, no further diversity loss took place in the A and B genomes during the polyploidy bottleneck accompanying the evolution of *T. aestivum *from domesticated tetraploid wheat [16]. Levels of diversity in the *T. aestivum *D genome are unknown.

Genetic evidence suggests that wild emmer was domesticated in the Diyarbakir region in southeastern Turkey [21,22]. The result was hulled domesticated emmer (*T. turgidum *ssp. *dicoccon*), which was then the primary source of free-threshing tetraploid wheat, such as durum (*T. turgidum *ssp. *durum*, henceforth *T. durum*). Transcaucasia and northwestern Caspian Iran appear to be the primary sites of the evolution of *T. aestivum *[23]. Gene flow from wild to domesticated tetraploid wheat and from tetraploid wheat and *Ae. tauschii *to *T. aestivum *has been experimentally documented [23-27] but its impact on the evolution of the *T. aestivum *A, B, and D genomes is not clear.

We report here the development of GSPs for *T. aestivum *and their use in sequencing of *T. aestivum *genes with the goal of characterizing the nucleotide diversity of the wheat genomes and discovering SNPs. To make the GSP development possible, a set of primers anchored in conserved exons flanking one or several introns was developed and is also reported. We refer to these as conserved primers (CPs), as in [13]. Primers of this type have also been known as conserved orthologous sets (COS) [28]. A map of genes bearing SNPs constructed in diploid *Ae. tauschii *is presented and compared with wheat deletion-bin gene maps [29]. Nucleotide diversity in individual chromosomes in a wild emmer population from the Diyarbakir region in Turkey and in *T. aestivum *was computed and the distribution of diversity among and within wild emmer and *T. aestivum *genomes was used to analyze the early stages of polyploid evolution.

## Results

### GSP development and SNP discovery

**Figure 1:**
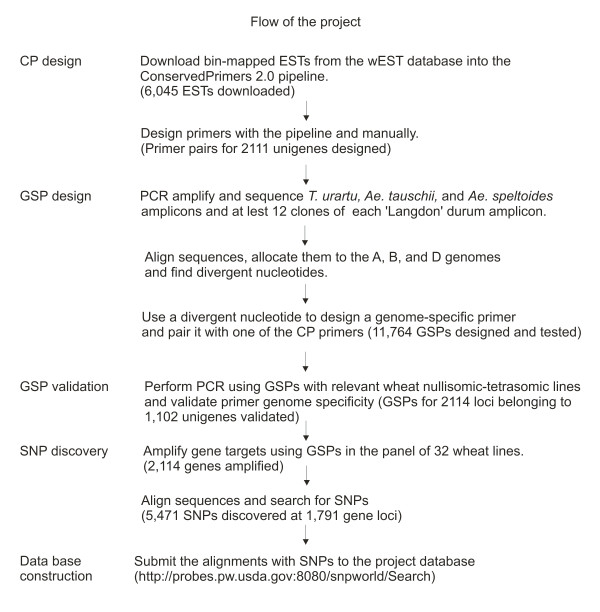
**Project flow chart**.

The process of GSP and SNP development is summarized in Figure 1. A total of 6,045 wheat ESTs was downloaded from the wEST database into the pipeline and CPs anchored in exons and flanking one or two introns were developed. The Southern hybridization profiles of the ESTs were examined in the wEST database http://wheat.pw.usda.gov/cgi-bin/westsql/map_locus.cgi and CPs for those that showed a complex profile were eliminated. Amplicons were obtained with CPs for 1,599 *T. urartu *genes, 1,583 *Ae*. s*peltoides *genes, and 1,574 *Ae. tauschii *genes and were sequenced. A total of 1,442 genes was cloned and sequenced from Langdon durum wheat. A total of 11,764 GSPs was designed and tested for genome specificity by PCR amplification of *T. aestivum *nullisomic-tetrasomic (N-T) lines. GSPs derived from 1,102 EST unigenes (705 in the A genome, 703 in the B genome, and 706 in the D genome) were validated by PCR with N-T lines.

Target DNA was PCR amplified in 32 wheat lines (Tables 1 and 2) using GSPs. A total of 41,065,555 bp of the amplicons was sequenced (14,734,124 bp in the A genome, 14,554,737 bp in the B genome, and 11,776,694 bp in the D genome) using GSP pairs as sequencing primers, and 5,471 SNPs at 1,791 loci were discovered.

**Table 1:** Lines of tetraploid and hexaploid wheat used for SNP discovery

Species	Database code	Line	Origin
*T. turg*. ssp. *dicoccoides*	Td01	PI 428020	Diyarbakir, Turkey
*T. turg*. ssp. *dicoccoides*	Td02	PI 428027	Diyarbakir, Turkey
*T. turg*. ssp. *dicoccoides*	Td03	PI 428053	Diyarbakir, Turkey
*T. turg*. ssp. *dicoccoides*	Td04	PI 428073	Diyarbakir, Turkey
*T. turg*. ssp. *dicoccoides*	Td05	PI 428064	Diyarbakir, Turkey
*T. turg*. ssp. *dicoccoides*	Td06	PI 428082	Diyarbakir, Turkey
*T. turg*. ssp. *dicoccoides*	Td07	PI 428083	Diyarbakir, Turkey
*T. turg*. ssp. *dicoccoides*	Td08	PI 428086	Diyarbakir, Turkey
*T. turg*. ssp. *dicoccoides*	Td09	G 2844	Diyarbakir, Turkey
*T. turg*. ssp. *dicoccoides*	Td10	G 2040	Diyarbakir, Turkey
*T. aest*. ssp. *aestivum*	Ta11	PI 166698	Turkey
*T. aest*. ssp. *aestivum*	Ta13	PI 166792	Turkey
*T. aest*. ssp. *aestivum*	Ta16	PI 622268	Iran,
*T. aest*. ssp. *aestivum*	Ta17	Yangxian Yangqianmai	Shaanxi (5660)
*T. aest*. ssp. *aestivum*	Ta18	Yecora Rojo	California
*T. aest*. ssp. *aestivum*	Ta19	PI 119325	Turkey
*T. aest*. ssp. *aestivum*	Ta20	PI 622233	Iran
*T. aest*. ssp. *aestivum*	Ta21	Chinese Spring	China
*T. aest*. ssp. *aestivum*	Ta23	Opata 85	CIMMYT
*T. aest*. ssp. *compactum*	Ta12	PI 166305	Turkey
*T. aest*. ssp. *compactum*	Ta14	PI 350731	Austria
*T. aest*. ssp. *compactum*	Ta15	PI 410595	Pakistan
*T. aest*. ssp. *spelta*	Ta22	405a (DV1132)	Iran

**Table 2:** Synthetic wheats used for SNP discovery

Name*	Database code	Source of AB genomes	Source of the D genome
ITMI synthetic^(1)^	Sn24	*T. t. durum *'Altar'	CIMMYT W7984, CIGM86.940, *Ae. tauschii *ssp. *strangulata*, (collected by H. Kihara near Mazadaran, Iran)
RL5402^(2)^	Sn25	TetraCantach	RL5261, *Ae. tauschii *ssp. *typica*
RL5403^(2)^	Sn26	TetraCantach	RL5266, *Ae. tauschii *ssp. *anathera*
RL5405^(2)^	Sn27	TetraCantach	RL5288. *Ae. tauschii *ssp. *strangulata*, (originally supplied by M. Tanaka as KUSE 2144)
RL5406^(2)^	Sn28	TetraCantach	RL5289. *Ae. tauschii *ssp. *meyeri*,
62052_4^(1)^	Sn29	*T. durum *'Croc_1'	CIMMYT 205, *Ae. tauschii *ssp. *tauschii* (PI452130, Hunan, China)
62056_4^(1)^	Sn30	*T. durum *'Croc_1'	CIMMYT 224 = CLAE 25, *Ae. tauschii *ssp.*tauschii *(collected by H. Kihara near Gilan, Iran)
161725_0^(1)^	Sn31	*T. durum *'Ceta'	CIMMYT 372 = CIGM86.940, *Ae. tauschii *ssp. *strangulata *(collected by H. Kihara near Kabul, Afghanistan)
Sear's synthetic^(3)^	Sn32	Unknown	Unknown

* Accessions designated with ^(1) ^were developed and supplies by A. Mujeeb-Kazi, at CIMMYT, those designated with ^(2) ^were developed and supplied by E.R. Kerber, Agriculture Canada, Winnipeg, Manitoba, and that designated with ^(3) ^was developed and supplied by E.R. Search, USDA-ARS, Columbia, Missouri

### SNP database

An online SNP database http://probes.pw.usda.gov:8080/snpworld/Search was constructed. It contains sequences of GSPs for the amplification and sequencing of 2114 loci and other relevant information about the ESTs and SNPs (such as deletion-bin mapping of each EST), top ten blast hits of each EST, alignments of nucleotide sequences generated with primers derived from each EST, a reference sequence for a locus and its source, and graphical and numerical displays of each SNP. Reference sequences were used to specify the positions of SNPs. For the majority of the loci, the cv 'Chinese Spring' (code Ta21, Table 1) sequence was used as a reference sequence because of the central position of Chinese Spring in the unrooted phylogenetic tree of 468 *T. aestivum *lines (Additional file 1, Figure S1). If the sequence from Chinese Spring was unavailable, the next most complete sequence for the locus was used. SNPs can be viewed in the context of the entire reference sequence in the expanded view window for each EST.

The database also contains data for portions of 1,651 genes amplified and sequenced with CPs in *T. urartu*, *Ae. speltoides*, and *Ae. tauschii *http://probes.pw.usda.gov:8080/snpworld/Search. The accession used as a reference sequence for a locus is indicated for each species. Data in the database include 488 polymorphic loci containing 1,271 SNPs for *T. urartu*, 463 polymorphic loci containing 1,218 SNPs for *Ae. speltoides*, and 641 polymorphic loci containing 2,203 SNPs for *Ae. tauschii*. Additional SNPs for *Ae. tauschii *can be found in the database for the D genomes of the synthetic wheats.

### Diversity maps

A single *Ae. tauschii *EST linkage map [30] was used as the backbone of the diversity maps. The *Ae. tauschii *map backbone contained 870 loci (Table 3). Cosegregating genes were allocated into "recombination blocks" which were sequentially numbered (Additional file 2, Table S1). The order of orthologous genes in rice was used to order genes within a recombination block.

**Table 3:** Loci mapped on the basis of linkage and synteny and the total number of EST loci with estimated diversity (Div. loci) on the map

Chromosome	Length (cM)	Total loci mapped	Linkage mapped loci	Synteny mapped loci	Div. loci
1	180.5	212	148	64	125
2	186.9	171	92	79	142
3	197.0	240	186	54	151
4	127.4	198	107	91	155
5	171.2	133	86	47	113
6	149.3	205	119	86	145
7	154.5	195	132	63	156

Total	1166.8	1354	870	484	987

Synteny of the *Ae. tauschii *genetic map with the rice genome sequence [30] was exploited in mapping additional loci for which the parents of the *Ae. tauschii *mapping population were not polymorphic (Table 3) and which met the conditions detailed in Materials and Methods (Map construction). In a few cases, in which an ambiguity was encountered in the rice genome sequence, sorghum and *Brachypodium distachyon *genome sequences were employed [30,31]. Consider, for example locus *BG313769 *located on the short arm of chromosome 1 D (Additional file 2, Table S1). This locus was mapped to bins 1AS1, 1BS9, and 1DS1 [32]http://wheat.pw.usda.gov/cgi-bin/westsql/map_locus.cgi. The locus with the highest sequence similarity in rice is on pseudomolecule Os5 starting at nucleotide 1,903,106. Os5 is homoeologous with 1DS and mapping of the locus in the 1AS1, 1BS9, and 1DS1 bins is consistent with the position of the locus in Os5 (Additional file 2, Table S1). PCR using genomic DNA of N1A-T1B and N1A-T1 D as templates with the *BG313769 *A-genome GSPs showed that the locus used for the diversity study was on chromosome 1A http://probes.pw.usda.gov:8080/snpworld/Search. Inserting locus *BG313769 *into the map on the basis of synteny of 1AS1, 1BS9, and 1DS1 with Os5 placed it between recombination block 36 (locus *BE445121*, which is at 56.85 cM on the *Ae. tauschii *map and at nucleotide 1,679,201 in the Os5 pseudomolecule) and recombination block 37 (locus *BF291549*, which is at 57.06 cM on the *Ae. tauschii *map and at nucleotide 1,954,380 in the Os5 pseudomolecule). Locus *BG313769 *and its diversity data were therefore placed between loci *BE445121 *and *BF291549*. No cM value was attached to the locus but its coordinates on Os5 were given (Additional file 2, Table S1).

Loci corresponding to 484 ESTs were inserted into the diversity maps on the basis of this process (Additional file 2, Table S1), bringing the total number of loci on the map to 1,354 (Table 3). Diversity was estimated from at least one genome for 987 EST loci on the map. From 348,938 to 351,542 bp were sequenced and mapped on the diversity maps for each genome × taxon combination (Table 4). The numbers of discovered SNPs ranged from 377 in the *T. aestivum *D genome to 1,979 in the wild emmer B genome. The highest average number of haplotypes per gene and highest average haplotype diversity was in the D genome of synthetic wheats whereas the lowest number of haplotypes per gene and lowest haplotype diversity was in the D genome of *T. aestivum *(Table 4). In wild emmer and *T. aestivum*, the average numbers of haplotypes per gene and haplotype diversity did not significantly differ between the A and B genomes (Table 4). However, both variables were significantly higher in the genomes of wild emmer than in the corresponding genomes of *T. aestivum *(Table 4).

**Table 4:** Nucleotides sequenced, SNPs discovered, average number of haplotypes (H), and haplotype diversity (h)

Species	Genome	*n*	**Nucl**.	SNPs	Nucl./SNP	*H*	*h*
*T. aestivum*	A	13	351542	966	364	1.82c*	0.22c
*T. aestivum*	B	13	348938	1008	346	1.72c	0.21c
*T. aestivum*	D	13	349748	377	927	1.23d	0.06d
*T. dicoccoides*	A	10	351542	1516	232	2.05ab	0.31b
*T. dicoccoides*	B	10	348938	1979	176	2.22a	0.33b
Synthetic 6*x *wheat	D	9	349029	1727	202	2.39a	0.47a

* Means followed by the same letter are not significantly different at the 5% probability level. Individual chromosome means (Table 9) were used as variables in ANOVA.

### Superimposition of diversity maps on the deletion-bin maps

Wheat EST deletion-bin maps are an important resource for the use of ESTs in wheat comparative mapping, map-based cloning of wheat genes, comparative genomics, and other genetic and genomic applications. To facilitate cross-referencing of EST diversity data developed here with EST deletion-bin maps, the wheat diversity maps were superimposed on the deletion bin maps (Additional file 2, Table S1).

The *Ae. tauschii *linkage map [30] and wheat deletion-bin maps share large numbers of loci, which facilitated comparison of the two sets of maps. Only loci mapped by linkage were used for these comparisons. Totals of 534, 654, and 646 ESTs on the wheat A-, B- and D-genome deletion-bin maps were compared, respectively. The bin location of a locus was considered incongruent between the genetic and deletion-bin maps if it disagreed with the order of recombination blocks (Additional file 2, Table S1); the order of loci within recombination blocks was disregarded. The known translocation differences involving chromosome 4A and chromosome arms 5AL and 7BS [33,34] were not considered. Because the genetic maps of *Ae. tauschii *chromosomes are highly colinear with the rice pseudomolecules (Additional file 2, Table S1) most of the disagreements between the linkage maps and deletion-bin maps would have to be due to structural differences between wheat and *Ae. tauschii *chromosomes or due to incompleteness or inconsistencies in the deletion-bin maps.

The *Ae. tauschii *linkage map portion of the diversity maps (Additional file 2, Table S1) is expected to be more consistent with the D-genome deletion-bin map than the A- and B-genome deletion-bin maps because the *Ae. tauschii *chromosomes are phylogenetically more closely related to those of the wheat D genome than to those of the wheat A and B genomes, and this was indeed observed. While the locations of only 8.8% of the loci on the D-genome deletion-bin maps were incongruent with the linkage map, 10.8 and 12.4% of the A- and B-genome loci were incongruent (Table 5). The greatest discrepancies relative to gene order in *Ae. tauschii *and rice were encountered in chromosome arms 1AL, 5AS, 7AL, 1BL, 5BS, 4DL, and 7DS, and none were found in chromosome arms 2BS, 2DS, 2DL, 3DS, and 5DL (Table 5 and Additional file 2, Table S1).

**Table 5:** Agreement between the locations of EST loci on the Ae. tauschii linkage map and wheat deletion-bin maps

	A genome		B genome		D genome	
**Chrom. arm**	**Total loci**	**% discordant loci**	**Total loci**	**% discordant loci**	**Total loci**	**% discordant loci**

1S	62	6.5	60	20.0	73	6.8
1L	38	31.6	49	20.4	48	16.7
2S	24	8.3	36	0.0	26	0.0
2L	39	2.6	36	5.6	37	0.0
3S	48	2.1	48	14.6	45	0.0
3L	82	1.2	92	8.7	97	9.3
4S	-*	-	27	14.8	29	10.3
4L	-*	-	55	1.8	59	27.1
5S	23	21.7	24	25.0	23	4.3
5L	39	12.8	37	10.8	33	0.0
6S	29	3.4	26	19.2	24	12.5
6L	53	9.4	59	11.9	48	8.3
7S	51	3.9	56	8.9	55	18.2
7L	46	23.9	49	12.2	49	10.2

Mean		10.8		12.4		8.8

* Chromosome 4A is extensively rearranged, and the congruence of the 4A deletion-bin map could not be meaningfully compared with the *Ae. tauschii *linkage map.

### Nucleotide diversity

From 609 to 704 genes with estimated diversity were mapped in a genome × species combination (Table 6). However, some of the loci were excluded from diversity analyses because of small sample size or because of unreasonably high diversity indicating the possibility of orthologous or paralogous sequences being included in a diversity estimate. The numbers of loci used for analyses of diversity were therefore lower (Table 6). Of the analyzed loci, 305 (52%) and 296 (51%) were polymorphic in the A and B genomes of *T. aestivum*, respectively, and 316 (54%) and 338 (59%) were polymorphic in the A and B genomes of wild emmer, respectively (Table 6). Only 138 (20%) loci of the 679 analyzed in the *T. aestivum *D genome were polymorphic (Table 6). Because the same GSPs resulted in the discovery of 477 (74%) SNP-bearing loci in the D genome of synthetic wheats (Table 6), the low number of polymorphic loci in the wheat D genome must be an attribute of wheat, not of *Ae. tauschii*, its diploid source.

**Table 6:** Numbers of loci on the diversity maps harboring one or more SNPs, the total numbers of loci with estimated diversity (nt), and the total numbers of loci used for analyses (na)

	*T. aestivum*			*T. dicoccoides*		Synthetic wheat
**Chromosome**	**A genome**	**B genome**	**D genome**	**A genome**	**B genome**	**D genome**

1	43	49	23	53	50	66
2	42	45	30	49	49	66
3	27	36	11	30	40	54
4	55	28	20	54	62	75
5	45	35	6	37	31	58
6	39	47	25	42	50	87
7	54	56	23	51	56	71

Total *	305a	296a	138b	316a	338a	477c

*n*_t_	619	609	704	619	609	704

*n*_a_	590	584	679	585	576	650

*Sums followed by the same letter are not statistically different at the 5% probability level.

Genome-wide *θ_w_*, and *θ_π _*were similar between the *T. aestivum *A and B genomes (Table 7). Both estimates were higher than those in the *T. aestivum *D genome (Table 7). The estimates were also similar between the A and B genomes in wild emmer, which showed higher diversity than the corresponding genomes in *T. aestivum *(Table 7).

**Table 7:** Total nucleotide diversity, diversity in coding sequences, noncoding sequences (introns and UTRs), replacement and silent codon positions

Genome	Total diversity (× 10^-3^)			Coding (× 10^-3^)		Noncoding (× 10^-3^)		Replacement (× 10^-3^)		Silent (× 10^-3^)		Ratio replac./silent	
	***θ_w_***	***θ_π_***	***D***	***θ_w_***	***θ_π_***	***θ_w_***	***θ_π_***	***θ_w_***	***θ_π_***	***θ_w_***	***θ_π_***	***θ_w_***	***θ_π_***

*T.a*. A	0.59c	0.57c	-0.09b	0.54b	0.55b	0.76bc	0.72bc	0.30b	0.30b	1.38bc	1.46a	0.25a	0.24a
*T.a*. B	0.56c	0.57c	0.00b	0.55b	0.59b	0.89bc	0.89b	0.32b	0.36b	1.40bc	1.44a	0.22a	0.22a
*T.a*. D	0.22d	0.18e	-0.57c	0.22c	0.18c	0.36d	0.32d	0.06c	0.06c	0.79d	0.59b	0.10b	0.11b
*T.d*. A	0.68b	0.70d	0.09b	0.58b	0.60b	0.91bc	0.98b	0.32b	0.36b	1.43bc	1.47a	0.23a	0.26a
*T.d*. B	0.76b	0.72cb	-0.12b	0.56b	0.55b	1.21b	1.17b	0.31b	0.30b	1.47b	1.46a	0.29a	0.29a
Synth. D	1.31a	1.51a	0.56a	1.13a	1.25a	1.84a	2.09a	0.52a	0.53a	3.35a	3.87d	0.17ab	0.16b

* Means sharing the same letter are not significantly different at the 0.05 probability levels.

Tajima's *D *contrasts *θ_w_*, and *θ_π _*to detect differences in the distribution of diversity relative to neutral expectations. The expectation for a neutral locus in a population is a Tajima's *D *of zero. Positive values of Tajima's *D *indicate a paucity of rare alleles and a preponderance of intermediate frequency alleles while negative values indicate a preponderance of rare alleles and a paucity of intermediate frequency alleles. Average Tajima's *D *was near zero in the A and B genomes of *T. aestivum *and wild emmer but was negative in the *T. aestivum *D genome and positive in the *Ae. tauschii *genome present in synthetic wheats (Table 7). The positive value of Tajima's *D *in the D genome of synthetic wheats is very likely due to strong subdivision of *Ae. tauschii *into two major subpopulations. This subdivision has been acknowledged taxonomically by elevating individuals of the two subpopulations to subspecies, *Ae. tauschii *ssp. *strangulata *and *Ae. tauschii *ssp. *tauschii *[35]. Estimates of diversity at the replacement to silent codon sites in the D genome were similar to those in *Ae. tauschii *and differed in both genomes from those in the A and B genomes of *T. aestivum *and wild emmer (Table 7).

### Diversity among individual chromosomes

**Figure 2:**
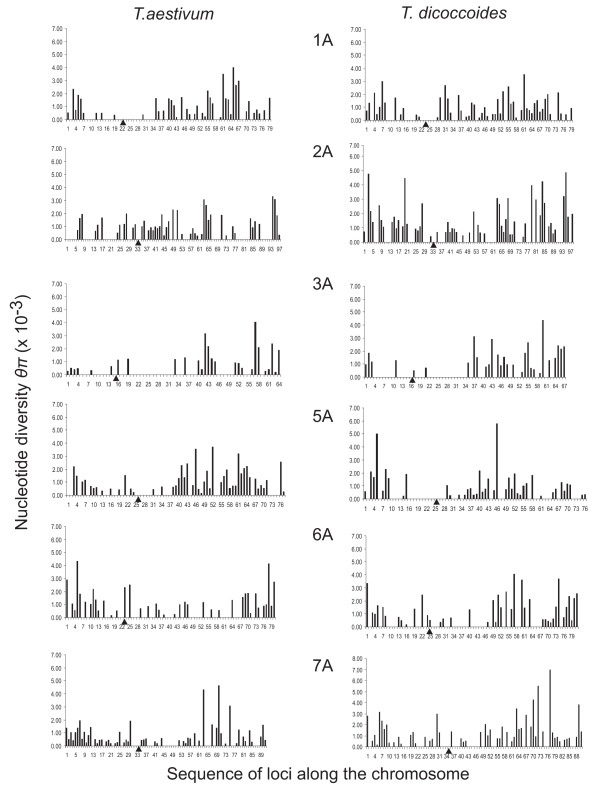
**Nucleotide diversity *θ_π _*of individual A-genome genes**. Gene diversity along the A-genome chromosomes in *T. aestivum *and wild emmer (*T. dicoccoides*) in the Diyarbakir region in Turkey. Chromosome 4A is excluded because the order of genes does not conform to the *Ae. tauschii *genetic map. Monomorphic loci are depicted with zero diversity. The gene order along the diversity maps in Additional file 2 Table S1 is used on the X-axis, and the maps are oriented with the most distal gene in the short arm to the left. Centromere is indicated by a triangle. Genetic distances between genes are not depicted.

In the A genome of wild emmer and *T. aestivum*, diversity was lower in chromosome 4A than in the remaining chromosomes (Table 8). This was true for diversity in coding sequences and in replacement and silent codon positions (Additional file 1, Tables S1, S2). Because chromosome 4A differs structurally from the *Ae. tauschii *homoeologue, the distribution of diversity along the chromosome was not investigated and it is not included in Figure 2 and Additional file 1, Figure S2, which illustrate the distribution of nucleotide diversity and the number of haplotypes per gene among and along the A-genome chromosomes. The distribution of diversity on chromosome 4A relative to its rearrangements will be addressed separately. In wild emmer, chromosome 5A also had lower diversity than the genome-wide average. Chromosome 5A of *T. aestivum *and chromosomes 2A and 7A of wild emmer had higher diversity than the genome-wide average. With the sole exception of *T. aestivum *chromosome 2A, diversity was low in genes in proximal chromosomal regions and high in genes in distal chromosomal regions (Figure 2). In *T. aestivum *chromosome 3A, most genes had only one or two haplotypes (Additional file 1, Figure S2). Average Tajima's *D *was close to zero in most A-genome chromosomes in *T. aestivum *with the exception of chromosome 7A, which had a negative average value, and chromosome 6A, which had a positive average value (Table 8). In wild emmer, chromosome 4A had a negative Tajima's *D *and chromosomes 2A and 7A had positive values.

**Table 8:** Average nucleotide polymorphism (θw), nucleotide diversity (θπ), and Tajima's D per chromosome

	*T. aestivum*									Wild emmer						Synthetic wheats		
	**A genome** **(× 10^-3^)**			**B genome** **(× 10^-3^)**			**D genome** **(× 10^-3^)**			**A genome** **(× 10^-3^)**			**B genome** **(× 10^-3^)**			**D genome** **(× 10^-3^)**		

**Chromosome**	***θ_w_***	***θ_π_***	***D***	***θ_w_***	***θ_π_***	***D***	***θ_w_***	***θ_π_***	***D***	***θ_w_***	***θ_π_***	***D***	***θ_w_***	***θ_π_***	***D***	***θ_w_***	***θ_π_***	***D***

*1*	0.67	0.64	-0.14	0.66	0.71	0.07	0.31	0.32	-0.10*	0.69	0.73	0.09	0.82	0.90	0.37	1.18	1.28	0.42
*2*	0.56	0.57	0.08	0.74*	0.87*	0.30	0.40	0.29	-0.65	0.83*	0.96*	0.50*	0.79	0.81	0.06	1.07*	1.23	0.57
*3*	0.49	0.46	-0.21	0.66	0.72	0.30	0.11*	0.08*	-0.79	0.60	0.65	0.21	0.81	0.76	-0.10	1.16	1.37	0.73
*4*	0.45*	0.42*	-0.11	0.24*	0.16*	-0.78*	0.15	0.10	-0.77	0.59	0.50*	-0.39*	0.75	0.61	-0.56*	1.19	1.41	0.61
*5*	0.77*	0.76*	0.15	0.45	0.48	0.21	0.09*	0.06*	-0.84	0.54*	0.51*	-0.18	0.56*	0.47*	-0.58*	1.65*	1.82*	0.39
*6*	0.60	0.66	0.26	0.65	0.66	0.06	0.21	0.21	-0.27	0.77	0.80*	0.05	0.79	0.76	-0.07	1.67*	1.90*	0.44
*7*	0.63	0.51	-0.57	0.66	0.59	-0.28*	0.24	0.16	-0.86	0.69*	0.77*	0.38*	0.77	0.74	-0.05	1.34	1.63	0.78*

Coef. variat.	0.18	0.21		0.30	0.38		0.52	0.59		0.15	0.23		0.12	0.19		0.18	0.18	

* Means outside of the 99% bootstrap confidence interval of the genome mean

**Figure 3:**
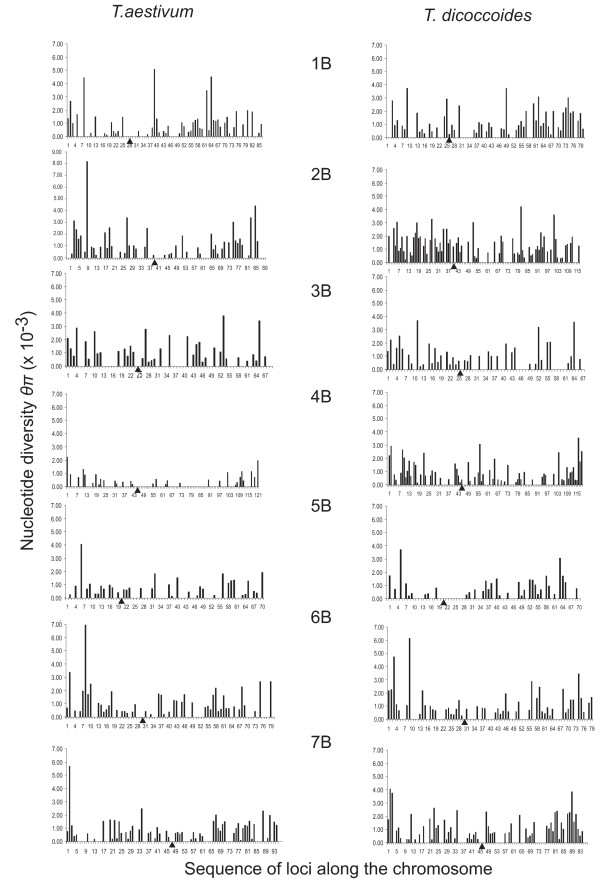
**Nucleotide diversity *θ*_π _of individual B-genome genes**. Gene diversity along the B-genome chromosomes in *T. aestivum *and wild emmer (*T. dicoccoides*) in the Diyarbakir region in Turkey. See Figure 2 for details.

In the B genome of *T. aestivum*, chromosome 2B had higher diversity and chromosome 4B had lower diversity than the rest of the chromosomes (Table 8). Diversity was low across the entire chromosome 4B (Figure 3), which also had the lowest number of haplotypes per locus and lowest haplotype diversity (Table 9). Except for genes in the distal region of the short arm of 4B, in which three haplotypes were observed in several genes, the proximal region of the short arm and the entire long arm had only one or two haplotypes per gene (Additional file 1, Figure S3). The sole exception to this trend in the entire long arm was locus *BF201102*, which had three haplotypes. However, the third haplotype caused by a singleton SNP that was not observed in any of the remaining 31 accessions could be a sequencing error. No other B-genome chromosome showed a similar pattern either in *T. aestivum *or wild emmer (Figure 3 and Additional file 1, Figure S3). Wild emmer chromosome 5B had reduced diversity, lower number of haplotypes per gene, and lower haplotype diversity compared to the genome mean (Tables 8 and 9). Diversity was reduced only in the proximal regions of both arms (Figure 3), and three or more haplotypes were observed in many genes (Additional file 1, Figure S3). As in the A genome, most chromosomes in both *T. aestivum *and wild emmer showed low diversity in the proximal regions (Figure 3). *T. aestivum *chromosome 4B and wild emmer chromosome 5B had highly negative average Tajima's *D*. In wild emmer, chromosome 4B also had a negative Tajima's *D *and a high ratio of silent to replacement sites (Additional file 1, Table S1).

**Table 9:** The average numbers of haplotypes per locus (H) and haplotype diversity (h)

	*T. aestivum*						*T. dicoccoides*				Synthetic wheats	
	**A genome**		**B genome**		**D genome**		**A genome**		**B genome**		**D genome**	

**Chrom.**	***H***	***h***	***H***	***h***	***H***	***h***	***H***	***h***	***H***	***h***	***H***	***h***

1	2.09*	0.29*	1.92*	0.26*	1.25	0.08*	2.13	0.30	2.17	0.36*	2.47	0.52
2	1.85	0.23	1.79	0.25	1.33*	0.11*	2.25*	0.33*	2.59*	0.34	2.40	0.47
3	1.65*	0.17*	1.78	0.24	1.13*	0.03*	2.03	0.30	2.11	0.32	2.26	0.41*
4	1.56*	0.17*	1.31*	0.07*	1.20	0.06	1.92	0.28	2.17	0.29*	2.24	0.43
5	2.04*	0.28*	1.68	0.21	1.11*	0.03*	2.01	0.31	1.99*	0.27*	2.43	0.50
6	1.71	0.22	1.86	0.25	1.27	0.08*	1.84*	0.27*	2.22	0.35	2.63*	0.50
7	1.84	0.18	1.74	0.20	1.31	0.06	2.14	0.35*	2.33	0.35	2.31	0.47

* Means outside of the 99% bootstrap confidence interval of the genome mean

**Figure 4:**
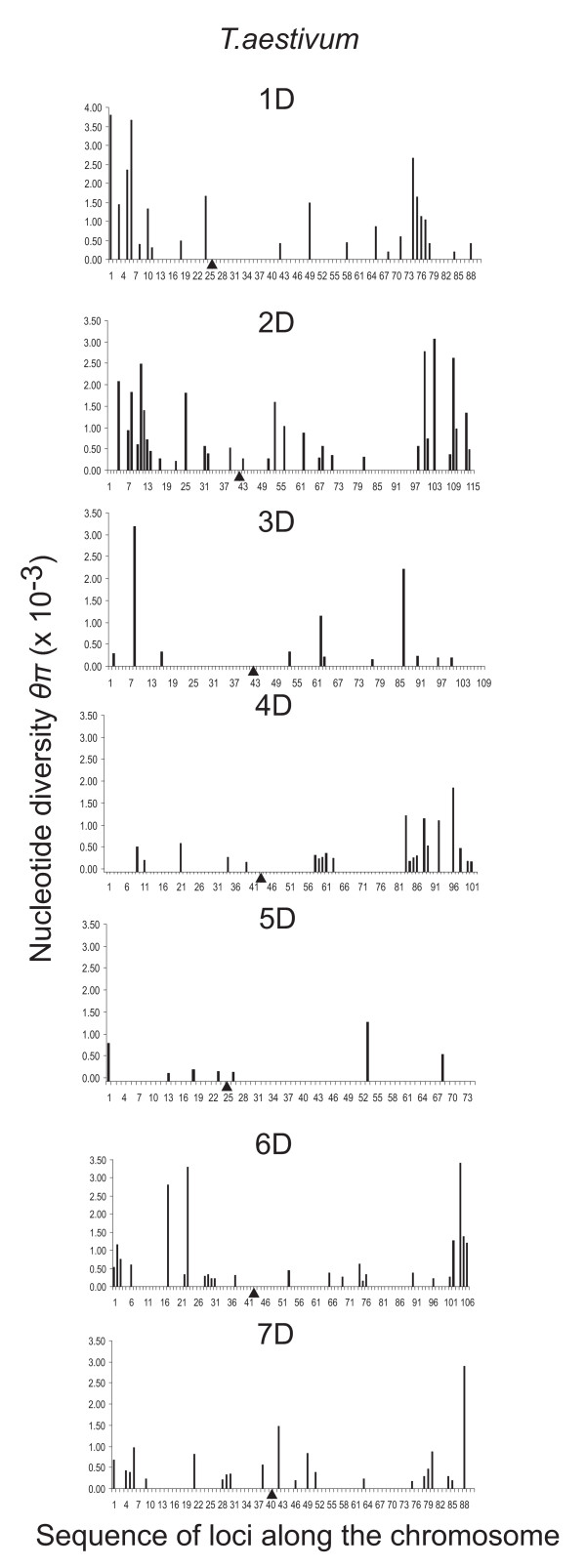
**Nucleotide diversity *θ*_π _of individual D-genome genes**. Gene diversity along the D-genome chromosomes in *T. aestivum*. See Figure 2 for details.

The D genome was the most uneven of the three *T. aestivum *genomes in terms of average nucleotide diversity per chromosome (Table 8). The coefficient of variation among the D-genome chromosomes was three times greater than in the D genome of synthetic wheats (Table 8). Nucleotide polymorphism *θ_w _*and nucleotide diversity *θ_π_*, the number of haplotypes per gene *H*, and haplotype diversity *h *were high in chromosomes 1 D and 2 D compared to genome averages (Table 9) and high values were distributed across the entire lengths of the chromosomes (Figure 4). Diversity was low in chromosomes 3 D and 5 D and both chromosomes were diversity impoverished across their entire lengths. Chromosome 4 D had low diversity across its length except for the distal region of the long arm in which diversity was high. A similar pattern was observed in chromosome 6 D, in which genes in both distal regions showed relatively high diversity and those in the proximal regions showed low diversity. In only a few genes were there more than two haplotypes (Additional file 1, Figure S4). Genes with more than two haplotypes were invariably in regions of elevated nucleotide diversity in the D genome. Computation of ratios of silent and replacement sites was greatly affected by the low levels of diversity in the D genome and was of limited value (Additional file 1, Table S2). All D-genome chromosomes had negative values of Tajima's *D *but all seven D-genome chromosomes in synthetic wheats had positive values of Tajima's *D *(Table 8), which, like the estimates of diversity, shows that negative Tajima's *D *is an attribute of the *T. aestivum *D genome but not of its ancestor.

Wall's *B *is a measure of intralocus linkage disequilibrium (LD). The higher the value of Wall's *B *the greater the proportion of neighboring sites in complete disequilibrium. In wild emmer and *T. aestivum*, the A and B genomes showed similar values of Wall's *B*, which ranged from 0.40 to 0.49 (Table 10). No significant differences were observed among the chromosomes. *Triticum aestivum *chromosome 7B and the low diversity *T. aestivum *chromosome 4B had the highest Wall's *B *values, 0.70 and 0.67, respectively, indicating that genes on those chromosomes have on average the highest levels of intralocus LD in the A and B genomes. The average Wall's *B *value of combined *T. aestivum *A and B genomes (0.49) was significantly higher (*P *= 0.024, paired *t*-test) than that of wild emmer (0.41), indicating a higher LD in *T. aestivum *than in wild emmer. In the D genome, average Wall's *B *(0.81) was significantly higher than in the A and B genomes indicating stronger linkage disequilibrium in the D-genome genes than in the A- and B-genome genes.

**Table 10:** Average values of Wall's B in the N number of genes

	*T. aestivum*		*T. dicoccoides*	
**Chromosome**	***N***	**Wall's *B***	***N***	**Wall's *B***

1A	18	0.29c*	15	0.31ab
2A	25	0.57ab	13	0.60a
3A	9	0.42bc	17	0.36ab
4A	12	0.82a	16	0.59a
5A	24	0.40c	15	0.19b
6A	20	0.45bc	14	0.35ab
7A	23	0.50bc	14	0.42ab

Mean		0.49a		0.40a
1B	25	0.32bc	23	0.44ab
2B	24	0.46ab	36	0.28a
3B	18	0.33bc	17	0.37ab
4B	9	0.67a	29	0.49b
5B	12	0.45ab	19	0.46ab
6B	26	0.42b	24	0.38ab
7B	27	0.70a	26	0.48ab

Mean		0.48a		0.41a
1D	11	0.88a		
2D	15	0.82a		
3D	2	1.00a		
4D	3	0.78a		
5D	2	1.00a		
6D	8	0.46b		
7D	6	0.75ab		

Mean		0.81b		

*Means followed by the same letter are not statistically different at the 5% probability level.

**Figure 5:**
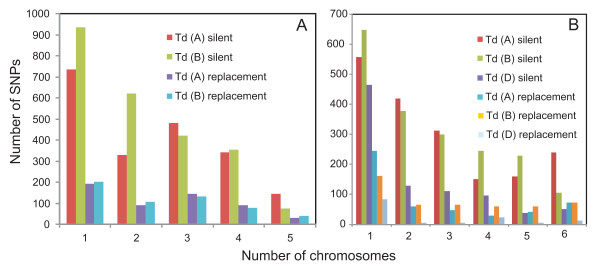
**The folded site frequency spectra**. Folded site frequency spectra of minor SNP alleles at silent and replacement positions in the A and B genomes, Td (A) and Td (B) respectively, in a sample of 10 homozygous accessions of wild emmer (*T. dicoccoides*). Each homozygous accession is equivalent to one chromosome. The plot depicts numbers of SNPs with the minor allele being observed in an indicated number of sampled chromosomes. (B) The folded site frequency spectrum of minor SNP alleles at the silent and replacement positions in the A, B, and D genomes, Ta (A), Ta (B), and Ta (D), respectively, in a sample of 13 homozygous accessions of *T. aestivum*. Each homozygous accession is equivalent to one chromosome. The plot depicts numbers of SNPs with the minor allele being observed in an indicated number of sampled chromosomes.

Minor allele frequency in the populations of 10 wild emmer chromosomes and 13 *T. aestivum *chromosomes giving rise to the folded site frequency spectra were computed for each polymorphic A, B, and D genome locus (Figure 5). Folded site frequency spectrum measures the number of times a SNP is observed in a sample. In both species, most minor alleles were present once. While the spectra were unimodal in *T. aestivum *they were bimodal in wild emmer, with the sole exception of the B-genome silent site spectrum. The difference between the spectra of the two populations suggests different demographic histories; the *T. aestivum *spectra resemble those typical of an expanding population whereas wild emmer spectra resemble a spectrum generated by a past bottleneck [36]. The folded spectra for the *T. aestivum *D genome declined faster than the spectra for the *T. aestivum *A and B genomes, which is consistent with higher numbers of rare polymorphisms in the D genome than in the A and B genomes as indicated by negative Tajima's *D *for the *T. aestivum *D-genome chromosomes.

## Discussion

### SNP discovery

A SNP discovery strategy based on the development of GSPs and their deployment in the search for SNPs in wheat is reported here. As a first step in SNP discovery, a CP pipeline was built starting with 6,045 ESTs [37]. For these ESTs and an additional 290 for which primers were generated manually, only a small portion, about 17.4%, ultimately resulted in validated GSPs. Even though the wheat genomes show low SNP levels, more than half of the A- and B-genome GSPs yielded an SNP, demonstrating that the development of GSPs is more difficult in polyploid wheat than SNP discovery.

The rationale for GSP development is that they make SNP markers versatile; any SNP detection method can theoretically be used if GSPs are available for the site in polyploid wheat. However, some SNP genotyping methods do not require prior PCR amplification of the SNP-containing targets in polyploid wheat [38,39] making GSPs superfluous. The cost/benefit ratio of GSP development should therefore be considered in the future development of SNPs for wheat.

Another aspect of the SNP development strategy employed here that needs consideration is the use of a distantly related relative as a source of information about the exon-splicing boundaries in ESTs for the design of CPs [37]. The reliance on wheat-rice comparisons preferentially selected for the conserved gene repertoire, which is concentrated in the proximal, low-recombination regions of wheat chromosomes [5,30,40-42]. There is also the potential that a focus on conserved loci could result in a downward bias in diversity estimates.

A focus on single-copy loci may also affect the distribution of loci with SNPs along the chromosomes. In wheat [40], as in other plants [43], single-copy genes are preferentially located in the proximal, low-recombination regions whereas distal, high-recombination regions are enriched for multigene families. Focusing on ESTs from single-copy genes may cause preferential development of SNP markers for genes located in the proximal, low-recombination regions of chromosomes.

For these multiple reasons, the SNP markers developed here are more abundant in the proximal, low-recombination regions of wheat chromosomes than in distal, high-recombination regions. This is particularly true for the distal 30 cM of the short arms of chromosomes in homoeologous groups 1, 2, and 3, which are poorly populated with SNP markers.

### Diversity maps

Comparative mapping based on RFLP markers showed that gene order along the *T. aestivum *homoeologous chromosomes is highly conserved and that any one chromosome of a trio of homoeologous chromosomes can be used to approximate gene order along the other two [44] and, as a matter of fact, along homoeologous chromosomes of other species throughout the tribe Triticeae [45]. Gene order is also surprisingly conserved across the entire grass family. Approximately 64, 65, and 66% of the loci on the *Ae. tauschii *genetic map are colinear with genes along the sorghum, *B. distachyon*, and rice pseudomolecules, respectively [30].

The conservation of gene order among wheat homoeologous chromosome and across the grass family was exploited here to summarize diversity in the wheat genomes using a single map. A comparative map of *Ae. tauschii *[30] was selected for that purpose. The high degree of gene synteny across grasses was exploited to insert into that map additional genes that in wheat contain SNPs but could not be mapped in *Ae. tauschii *for lack of polymorphism.

The utility of the *Ae. tauschii *linkage map as a representation of the linear order of genes in the wheat genomes depends on the extent to which the assumption of colinearity of the *Ae. tauschii *and wheat chromosomes is true. Known translocations exist among chromosomes 4A, 5A, and 7B, and chromosome 4A also acquired pericentric and paracentric inversions [33,34]. For chromosomes 4A and the translocated regions of 5A and 7B, the diversity maps reported here are of limited relevance.

Since virtually all of the ESTs employed in SNP discovery here had been previously mapped on the wheat deletion-bin maps, this is the first time it is therefore possible to compare the wheat bin maps with a high density genetic map of a closely related genome. The *Ae. tauschii *genetic map that formed the backbone of the diversity maps was highly colinear with rice, *B. distachyon*, and sorghum genomic sequences [30,31]. There was a remarkably good agreement between the deletion-bin maps and the *Ae. tauschii *genetic map for most chromosome arms and discrepancies were found for less than 10% of the loci. Some of these discrepancies were biological in nature. The greatest number of discrepancies was in the B-genome deletion-bin map and the smallest in the D-genome deletion-bin map. The numbers of paralogous loci in the B genome outnumber those in the A or D genomes 2 to 1 [41]. The B genome is also more prone to translocation [46-48] and undoubtedly other structural changes. Both paralogous gene duplications and changes in chromosome structure manifest themselves as breaks in synteny between the *Ae. tauschii *genetic map and wheat deletion-bin maps. The poorest fit between the genetic map and the deletion-bin maps found here for the B genome is therefore consistent with greater divergence of the B genome relative to the A and D genomes.

Although the wheat D-genome map was the most similar to the *Ae. tauschii *map of the three wheat deletion-bin maps, it too showed discrepancies relative to the *Ae. tauschii *map in several chromosome arms. The largest number of loci showing a perturbed location on the deletion-bin map was observed in chromosome arm 4DL. Ordering of loci in the 4DL arm bins on the basis of the *Ae. tauschii *genetic map resulted in interdigitation of loci mapped in the neighboring bins 4DL12 and 4DL13. The *Ae. tauschii *genetic map shows many rearrangements in that region compared to rice chromosome Os3 [30]. It is therefore possible that chromosome 4 D may contain a paracentric inversion spanning the boundary of bins 4DL12 and 4DL13, which could account for the difficulties encountered during an attempt to recombine wheat homoeologous chromosome arms 4DL and 4BL in the *KNA1 *region [49].

A total of 36% of the loci on the diversity maps was mapped on the basis of synteny with rice. Even though mapping of these loci was based on several lines of corroborating information, it is nevertheless an inference and must be treated with caution. The prerequisite corroborating information was not available for the remaining 209 (11.7%) of the A-, B-, and D-genome loci harboring SNPs, and these markers were neither included on the diversity maps nor used in computations of diversity estimates, although they were included in the database http://probes.pw.usda.gov:8080/snpworld/Search. The most frequent reason for the inability to map a locus on the basis of synteny was the failure to identify an orthologous region in rice. Synteny is more rapidly lost in the distal regions of wheat chromosomes due to greater rates of gene deletions and gene duplications in the distal regions than in the proximal regions [5,40,41]. This factor contributed to the poor SNP marker coverage in the distal regions of some of the chromosomes. For the same reasons, however, ESTs harboring SNPs that could not be mapped on the basis of synteny are preferentially located in distal chromosome regions. The project SNP database should therefore be interrogated if additional SNPs are needed, particularly those in the distal chromosome regions.

### Genetic application of the diversity maps

The diversity maps reported in Additional file 2, Table S1 provide a convenient summary of SNPs http://probes.pw.usda.gov:8080/snpworld/Search in genes that were mapped on the *Ae. tauschii *map. A *θ_w _*value of zero indicates no SNP was present and high values suggest several SNPs at a locus in the respective population of *T. aestivum *and wild emmer lines. Negative Tajima's *D *values indicate low frequency SNPs and positive Tajima's *D *values indicate a predominance of intermediate frequency SNPs at a locus.

Tetraploid wheats were parents of nine synthetic wheats that were screened with data subsequently reported in the SNP database. They included durum lines (Sn24, Sn29, Sn30, and Sn31), the tetraploid component of *T. aestivum *'Canthatch' (Sn25 to Sn28), and an emmer line (Sn31). SNPs present in these lines are tabulated in the database. Because they were not used in the computation of diversity measures, *θ_w _*may be 0.00 for a gene in Additional file 2, Table S1 but SNPs may exist in the A and B genomes of synthetics wheats in the database. This fact should be kept in mind when a specific locus is interrogated for a SNP on the diversity maps.

Although synthetic wheats RL5402, RL5403, RL5405, and RL5406 share tetraploid Canthatch as the source of their A and B genomes, they are occasionally polymorphic in the database. The tetraploid Canthatch was developed by recurrent backcrossing of the pentaploid hybrid *T. durum *'Steward' × *T. aestivum *'Canthatch' to *T. aestivum *Canthatch selecting tetraploid offspring in each generation [50]. SNPs occasionally observed among the four synthetic wheats are presumably residual germplasm of *T. durum *Steward present in the tetraploid Canthatch, indicating that a complete extraction of hexaploid wheat A and B genomes was not reached in tetraploid Canthatch and that the tetraploid is heterozygous at some loci.

### Sampling nucleotide diversity for SNP development

Nucleotide diversity measured as *θ_π _*was similar in the *T. aestivum *A and B genomes and averaged 0.59 × 10^-3^, which is close to an estimate of 0.8 × 10^-3 ^reported earlier [16]. The agreement between these two independent studies suggests that the sample of the *T. aestivum *lines used here was representative of *T. aestivum *and was adequate for SNP discovery in all three wheat genomes. However, nucleotide diversity averaged across genomes of wild emmer (*θ_π _*= 0.72 × 10^-3^) was lower than the estimated *θ_π _*= 2.7 × 10^-3 ^for wild emmer as a whole [16] indicating that the population in the Diyarbakir region has low diversity relative to species-wide samples of wild emmer. This is consistent with earlier RFLP results [22], which indicated that the greatest diversity in wild emmer exists in northern Israel, southern Lebanon, and southwestern Syria [22]. Because *T. aestivum *originated in Transcaucasia [35], the failure to sample wild emmer in those regions may have had a limited effect on the discovery of SNPs relevant for hexaploid wheat. However, it must have had a great effect on the discovery of SNPs relevant for durum wheat, because durum wheat originated in the eastern Mediterranean [22]. Inclusion of only a few durum accessions in the sample screened for SNPs here was inadequate to characterize durum diversity, and an additional SNP search is needed for cultivated tetraploid wheat.

### Wheat diversity architecture

In spite of the fact that the three *T. aestivum *genomes have coexisted within a single nucleus since the origin of *T. aestivum*, profound differences were found among them. The A and B genomes are more diverse and show more uniform distributions of diversity across the genome than does the D genome. Because of the short time that has elapsed since the origin of *T. aestivum*, 8,500 years or less [10], it is unlikely that most SNPs observed in *T. aestivum *originated there. It is much more likely that SNPs were contributed by gene flow from the ancestral species, tetraploid wheat and diploid *Ae. tauschii*, or potentially polyploid species of Aegilops having a D genome, such as *Ae. cylindrica*, that occasionally hybridize with wheat [51,52].

This intuitive argument is supported by differences in the ratio of replacement to silent polymorphisms in the *T. aestivum *genomes. Evolution in young polyploids is accompanied by relaxed purifying selection acting on genes, which is shown by an order of magnitude greater rate of fixation of deletions of single-copy genes in tetraploid wheat than in diploid *Ae. tauschii *and *T. urartu *[5]. If SNPs observed in *T. aestivum *were contributed by gene flow, genes in the A and B genomes should show ratios of replacement to silent site variation shifted towards 1.0 (indicating relaxed selection) compared to those in the D genome, which was observed. Additionally, if the haplotypes present in *T. aestivum *were largely contributed by gene flow, this could increase the effective population size *Ne *of the A and B genomes relative to the D genome because haplotype recombination in the A and B genomes could have taken place during the evolution of wild emmer. Hence LD in the A and B genomes of *T. aestivum *is expected to be stronger than in the A and B genomes of wild emmer and LD in the D genome of *T. aestivum *is expected to be stronger than in the *T. aestivum *A and B genomes, which is what was observed. We therefore conclude that most of the differences in diversity between the A and B genomes on the one hand and the D genome on the other hand can be attributed to differences in gene flow.

The difference in gene flow among the genomes has a material basis. It is well known that very little reproductive isolation exists between hexaploid and tetraploid wheat because these species readily hybridize and the resulting pentaploid hybrids are usually fertile [53]. In contrast, hybridization between hexaploid wheat and *Ae. tauschii *is difficult and hybrids are sterile [54]. Landraces of hexaploid and tetraploid wheat have often been grown together, which has facilitated hybridization. In contrast, sympatry between *T. aestivum *and *Ae. tauschii *has been limited by the geographic distribution of *Ae. tauschii*. Greater gene flow from the *T. aestivum *ancestors into the A and B genomes than into the D genome is therefore expected.

This study substantiated a previous survey of modern wheat varieties with SNPs developed here [55] and showed that limited gene flow into the *T. aestivum *D genome has enriched it for rare alleles. The preponderance of rare alleles in the D genome is indicated by the negative average Tajima's *D *observed in all seven D-genome chromosomes. Site frequency spectra in the *T. aestivum *genomes show a steeper decline in the D genome than in the A and B genomes, which is consistent with more limited gene flow into the *T. aestivum *D genome than into the A and B genomes. These observations agree with previous isozyme, RFLP, and SNP studies on the origin of hexaploid wheat, which suggested that wheat originated via a very limited number of hybridization events [23,24,26,56-58]. SNP data generated here showed that 93% of the 138 polymorphic genes in the D genome include only two haplotypes.

Diversity contributed by gene flow into wheat was further shaped by several factors. One was reduced effective recombination accompanying self-pollination, the prevalent mating system in wheat. Self-pollination can reduce the effective population size to half that expected under cross-pollination [59] and enhance the effects of genetic drift on diversity [60]. Self-pollination, by greatly impacting effective recombination [59], increases the sizes of chromosomal segments hitchhiking along with positively selected genes [61-64]. Low effective recombination is likely one of the contributing factors of the greatly uneven distribution of diversity in the *T. aestivum *D genome compared to the A and B genomes; the average *θ_π _*per chromosome was found to be six-fold higher in the most-diverse D-genome chromosome compared to the least-diverse D-genome chromosome. Diversity is high along the entirety of chromosomes 1 D and 2 D, the distal portion of the long arm of chromosome 4 D, and both distal regions of chromosome 6 D. In contrast, the entirety of chromosomes 3 D and 5 D, three-quarters of chromosome 4 D, and proximal regions of 6 D have very low levels of diversity. This suggests that under limited gene flow and self-pollination, genetic drift and selection may impact diversity along large chromosomal regions in wheat.

Several A- and B-genome chromosomes show that effects shaping the diversity of entire chromosomes may occasionally take place even under the regime of moderate gene flow in polyploid genomes. Diversity in *T. aestivum *chromosome 4B mimics in all respects diversity in the D genome. The entire chromosome is diversity impoverished and the chromosome has a highly negative Tajima's *D*. As in the D-genome chromosomes, most of the 4B genes have either one or two haplotypes. Chromosome 4B is polymorphic for a pericentric inversion in *T. aestivum *[65], and homoeologous group 4 has a lower number of genes than the remaining six Triticeae homoeologous groups [29], presumably due to the translocation of the gene-rich terminal region of the short arm of chromosome 4 to the long arm of chromosome 5 [30]. Recombination takes place primarily in genes. Low number of genes on chromosome 4B would probably result in low crossover frequencies in this chromosome, which was observed [66]. The net effects of limited effective recombination may be that a large portion of this chromosome has hitchhiked during episodes of positive selection during the evolution of *T. aestivum *or was subjected to a reduction in effective population size during episodes of background selection [60]. A long-range loss of diversity may have also taken place in wild emmer chromosome 5B, which also has a negative average Tajima's *D*. Another chromosome in which a chromosome-sized loss of diversity has taken place is 4A. In this chromosome, the loss of diversity was undoubtedly caused by the fixation of inversions suppressing recombination in a heterozygous state.

Another factor that must have had a significant impact on the architecture of diversity in wheat is the expression of the *Ph1 *locus, which is unique to polyploid wheat. Its primary function is to preclude recombination between homoeologous chromosomes [67-69]. Importantly, *Ph1 *also negatively effects recombination between heterozygous homologues [66]. The activity of *Ph1 *therefore has similar effects on diversity as self-pollination. For an unknown reason, *Ph1 *negatively affects recombination in the B genome more than in the A genome [66]. The *T. aestivum *B genome shows greater variation in diversity among chromosomes than the A genome. The coefficients of variation were 0.18 and 0.21 for *θ_w_*, and *θ_π _*among the *T. aestivum *A-genome chromosomes but were respectively 0.30 and 0.38 among the *T. aestivum *B-genome chromosomes, which is consistent with more reduced recombination in the B genome than in the A genome due to *Ph1 *effects. Recombination between the *Ae. tauschii *chromosomes and wheat D-genome chromosomes is even more affected by *Ph1 *than recombination between wheat heterozygous homologues [70]. In agreement, *T. aestivum *D-genome chromosomes show the greatest variation in diversity among the three genomes; coefficients of variation were respectively 0.52 and 0.59 for *θ_w_*, and *θ_π _*among the D-genome chromosomes. We suggest that the synergy of self-pollination and suppression of recombination due to *Ph1 *results in high levels of random drift, loss of diversity from large chromosome regions, and relatively high variance in diversity among chromosomes.

## Conclusions

Distinctly different diversity patterns were found in two closely related polyploid species of differing age, the recently evolved *T. aestivum *and the older wild emmer. In wild emmer, diversity is uniform among genomes and chromosomes but in *T. aestivum*, diversity is heterogeneous both among both genomes and chromosomes. These observations suggest the following scenario of polyploid evolution. In a nascent polyploid, diversity almost entirely depends on gene flow from the ancestral species. During that period, diversity is greatly affected by stochastic and directional processes, particularly under self-pollination that is wide spread in polyploids. Dependence on gene flow and the synergy of self-pollination and action of *Ph1-*like genes results in low and heterogeneous diversity across genomes. If gene flow cannot keep pace with the population expansion, diversity is dominated by rare alleles. Large chromosomal regions or whole chromosomes are subjected to genetic drift and hitchhiking resulting in their low diversity. As time passes, the accumulation of new mutations results in an increased and more uniformly distributed diversity across the genome, as is seen in wild emmer.

## Methods

### CP design

ESTs showing simple cDNA hybridization profiles with *T. aestivum *genomic DNA in Southern blots http://wheat.pw.usda.gov/cgi-bin/westsql/map_locus.cgi were selected from the wEST database [71] for CP design. Only ESTs mapped on the wheat deletion bin maps [32,72-77] were used. The wheat deletion-bin maps were constructed by hybridization of random cDNA clones with DNAs of 101 deletion stocks [78] and a set of wheat telocentric stocks [79] that subdivided the 21 wheat chromosomes into 159 bins [80]. CPs located in exons flanking one or more introns were designed on the basis of comparison of wheat EST sequences with rice genomic sequence. EST contigs or EST singletons were extracted from the wEST database http://wheat.pw.usda.gov/cgi-bin/westsql/map_locus.cgi and compared with rice genomic sequences to identify exon/exon junctions. The Primer3 program [81] was modified for PCR primer design in batch mode [82]. A pipeline for batch homology search between wheat ESTs and rice genomic sequence http://avena.pw.usda.gov/SNP/new/bioinformatics.shtml was built [37,82]. With this pipeline, PCR primers were successfully designed for 2,223 EST unigenes, 1,958 EST contigs and 265 EST singletons. Of these, primer pairs for 1,624 were from 5' ESTs and 599 from 3' ESTs. Since these primers were located in exons and were designed on the basis of homology with the rice exonic sequences, they were highly conserved in grasses; hence their name. An additional 290 primers for loci in the distal bins were designed manually using the Primer3 program or the GeneTools primer design program [83].

### GSP design

Genomic DNAs of *T. urartu *accessions G1812 (PI 428198, Turkey) and ICTW600161 (Syria, supplied by J. Valkoun, ICARDA, Syria), *Ae. tauschii *ssp. *strangulata *accession AL8/78 (Armenia, supplied by V. Jaaska, Estonian University, Tartu), and *Ae. tauschii *ssp. *tauschii *accession AS75 (Shaanxi, China) (Table 11) were used as PCR templates. Pairs of accessions were selected among 193 and 188 accessions representative of the geographic distribution of *T. urartu *and *Ae. tauschii¸ *respectively, and genotyped with random restriction fragment length polymorphism (RFLP) markers [23]. A pair of genetically distant accessions was selected within each species. Target DNAs were PCR amplified using CPs and amplicons were directly sequenced, using CPs as sequencing primers. *Triticum uratu *and *Ae. tauschii *are self-pollinating species, and the four accessions were assumed to be homozygous at the targeted loci but *Ae. speltoides *is cross-pollinating, and it was expected to be heterozygous at many loci targeted for sequencing. DNA of two randomly selected *Ae. speltoides *F_4 _plants from the cross 2-12-4 × PI 136909-12-II/134-1 [84] were therefore used as PCR templates in the hope that at least one was homozygous at a targeted locus and the amplicon could be sequenced.

**Table 11:** Diploid and tetraploid species used for the development of genome-specific primers

Species	Database code	Genome	Line	Origin
*T. urartu*	Tu01	A	PI428198 = G1812	Turkey
*T. urartu*	Tu02	A	ICTW600161 (ICARDA)	Syria
*Ae. speltoides*	As01	S	F_4 _/2-12-4//PI136909-12-II/134-1	Experimental line
*Ae. speltoides*	As02	S	F_4 _/2-12-4//PI136909-12-II/134-2	Experimental line
*Ae. tauschii*	At01	D	AL8/78	Armenia
*Ae. tauschii*	At02	D	AS75	China
*T. turgidum *ssp. *durum*	Lgd1 through 12	AB	Langdon, 12 clones	Cultivar

B-genome sequences were obtained from 'Langdon' durum wheat by PCR amplification of Langdon genomic DNA using CPs. The amplicons were purified using the Promega PCR amplicon purification kit and cloned to the TA cloning site of the pGEM-T vector (Promega) following manufacturer's recommendations, and after transformation, *E. coli *cells were plated on LB agar medium. Twelve positive transformants were picked and plasmid inserts were PCR amplified using M13-48 and T7 Universal Primers. The PCR reaction consisted of 1X Taq polymerase buffer, 0.2 mM dNTPs mix, 50 pmols of primers, 1U of Taq polymerase, and sterile distilled water. PCR conditions were 10 min at 94°C, 10 cycles of 94°C for 20 sec, 58°C for 20 sec, and 72°C for 2 min, followed by 35 cycles at 94°C for 20 sec, 55°C for 20 sec, and 72°C for 2 min. PCR was terminated by final extension at 72°C for 5 min. Success of PCR amplification was checked by 1% agarose electrophoresis. For sequencing, 5μl of amplified DNA was treated with exonuclease I (USB) and shrimp alkaline phosphatase (USB) according to manufacturer's recommendations in a 10μl reaction volume. The reaction was diluted to 18μl with water before an aliquot was taken for subsequent sequencing. The clones were sequenced as described below. The sequences of the clones were compared with the *T. urartu *and *Ae. speltoides *sequences and each Langdon clone was assigned to either the A or B genome. Because CPs annealed to both A- and B-genome templates during PCR amplification of Langdon DNA, chimeric amplicons could be generated during the amplification process [85]. A-genome/B-genome chimeric clones were occasionally encountered and attention was paid to their presence during the assignment of sequences to genomes.

The *T. urartu*, *Ae. speltoides*, *Ae. tauschii*, and Langdon sequences were assembled into contigs with the Staden program [86]. Clean FASTA sequences were aligned with ClustalW [87] or MUSCLE [88] programs. Sequence alignments were visually checked using Bioedit (Tom Hall, Ibis Therapeutics, Carlsbad, CA). Genome-specific nucleotide substitutions were recorded and GSPs were designed. The primers were limited to the Tm range of 55 to 60°C, so that subsequent PCR amplifications could be performed in batches. Genome-specific nucleotide substitution was used as the 3' end of each GSP [89]. In addition, the third nucleotide from the 3' end was occasionally purposely mismatch with the template to increase the genome specificity of the primer. These modifications are included in the GSPs reported in the SNP database http://probes.pw.usda.gov:8080/snpworld/Search. A GSP was combined with one of the CPs to obtain a primer pair for genome-specific amplification. Most of the amplicons therefore consisted of exonic and intronic sequences.

### GSP validation

Because the bin location of each targeted gene was known, only the chromosomes of the homoeologous group in which the bins were located were used for PCR validation of GSPs. DNA was PCR amplified from the relevant N-T in the *T. aestivum *'Chinese Spring' genetic background [90]. If a GSP functioned properly, DNA of the N-T line nullisomic for the chromosome in the targeted genome produced no amplicon but DNA of the N-Ts for the remaining two chromosomes of the homoeologous group produced amplicons. Primers that produced amplicons with DNA of all three N-Ts failed the validation step. This could happen if the gene was actually located in a different homoeologous group than assumed. Primers that failed the N-T test were therefore used in PCR with N-Ts for all 21 wheat chromosomes. If amplification occurred in all but one N-T, it was assumed that the targeted gene was located on the chromosome that was absent in the N-T line that failed to produce an amplicon. Such primers were considered validated. If none of the N-Ts consistently showed absence of an amplicon in one of the N-T lines, the putative GSP was discarded.

### SNP discovery

To maximize the likelihood of the relevance of discovered SNPs to cultivated wheat while minimizing the number of lines screened for SNPs, a resequencing panel representative of lines of wild emmer from the Diyarbakir region (10 lines) and *T. aestivum *(13 lines) (Table 1) was used. Twelve of the 13 *T. aestivum *lines were selected from representative branches of a neighbor-joining tree constructed for 476 *T. aestivum *lines genotyped at 153 RFLP loci [91] (Additional file 1, Figure S1). One *T. aestivum *line ('Opata 85') was added because it was one of the parents of the International Triticeae Mapping Initiative (ITMI) mapping population (Table 1) [92]. The wild emmer lines were selected from wild emmer populations in the Diyarbakir region so that each represented a branch in a neighbor-joining tree (Additional file 1, Figure S5) based on genetic distances using 131 RFLP loci [22]. In addition, 9 synthetic hexaploid wheats produced by crossing tetraploid wheat with *Ae. tauschii *and doubling the chromosome number [12] were included in the screening population. Synthetic wheat is used in wheat breeding as a source of new D-genome variation [93-95]. Four synthetics (Sn25 through Sn28, Table 2) selected for the project were supplied by E.R. Kerber (Agriculture Canada, Winnipeg). They were included because they were previously used as sources of *Ae. tauschii *chromosomes in the development of disomic substitution lines of single *Ae. tauschii *chromosomes in the Chinese Spring genetic background (J. Dvorak, unpublished). The donor of the A and B genomes of these synthetic wheats was a tetraploid extraction of *T. aestivum *'Canthatch' [50]. Synthetic Sn24 was the parent of the ITMI mapping population [92]. Synthetic wheats Sn29 through Sn31 were extensively used in the CIMMYT wheat breeding program. Finally, synthetic wheat Sn32 was the parent of a RFLP mapping population [44]. The tetraploid parents of Sn24, Sn29, Sn30, and Sn31 were durum and that of Sn32 was emmer. The *Ae. tauschii *parents of the synthetics, if known, are indicated in Table 2.

Target DNAs of these 32 lines were amplified with GSPs, the sequences were aligned and edited as described above and SNPs were submitted to the central database http://probes.pw.usda.gov:8080/snpworld/Search. All wheat sequences were also submitted to NCBI. Their accession numbers are HQ389550 to HQ391340.

### DNA sequencing

As explained above, a GSP primer pair consisted of a CP and a GSP primer. PCR amplification was performed in seven different labs but sequencing of the amplicons was performed centrally at the Western Regional Research Center, USDA-ARS, Albany, California. Two replicas containing 96 different processed amplicons were made in semi-skirted 96-well PCR plates. To make the first replica, 3 μl of a processed amplicon and 1μl of the corresponding CP primer (3.2 pmol/ul) were placed into a well of one plate. To make the second replica, 3 μl of a processed amplicon and 1μl of the corresponding GSP primer (3.2 pmol/ul) were placed into the corresponding well of a second plate. The plates were frozen and shipped on dry ice to the sequencing lab along with a directory of the amplicons in each well.

The plates were thawed in the sequencing lab and 1.5μl of 5X sequencing buffer, 1μl of 50% DMSO, 1μl of Big Dye v.3.1, and 2.5μl of deionized water were added to each well. The cycling conditions were: 5 min at 98°C followed by 40 cycles at 10 sec at 96°C, 5 sec at 50°C, and 4 min at 60°C. DNA was precipitated with ethanol followed by a 70% ethanol rinse, dried, 12 μl of sequencing grade formamide was added, and the DNA was sequenced on an ABI3730xl. Both strands of each amplicon were sequenced; one was produced in the plate containing CPs as sequencing primers (1^st ^DNA strand) and the other was produced in the plate containing GSPs as sequencing primers (2^nd ^DNA strand).

The Phred/Phrap [96] or Staden package http://staden.sourceforge.net/ programs were used for base calling and assembly of sequencing trace files. Assembled contigs were edited with the Staden package. Perl and Java programs were written to manipulate the data. The PolyPhred v. 5.0 program [97] and mutation detection modules from the Staden package were utilized for SNP detection.

### Map construction

A genetic map based on segregation of markers in a population of 572 F_2 _plants from the cross *Ae. tauschii *AL8/78 × *Ae. tauschii *AS75 [30] was used as a backbone for the development of diversity maps. The backbone map contained 878 markers of which 863 were ESTs; 12 of the remaining loci were random RFLP markers and three were microsatellite loci. ESTs were mapped either on the basis of RFLP or SNP. The latter were mapped with the SNaPshot™SNP assay (Applied Biosystems, Foster City, California) or GoldenGate BeadArray SNP assay (Illumina Inc., SanDiego, California). A total of 174 F_2 _plants was used for RFLP and SNaPshot mapping and 560 F_2 _were used for Illumina GoldenGate assays.

The EST markers were compared with the NCBI rice genomic sequence to assess the wheat-rice macrosynteny (henceforth synteny) [30]. Loci that cosegregated were grouped into "recombination blocks" [30] within which they were arranged to parallel the order of orthologous genes in rice. Genes that could not be mapped because of the lack of polymorphism between the parents of the *Ae. tauschii *mapping population were inserted into the *Ae. tauschii *map at a location corresponding to that of a putative rice orthologue, provided that the following conditions were met: (1) the allocation of the gene to wheat chromosome by PCR using N-T lines and GSPs agreed with the previous deletion-bin mapping, (2) the section of the wheat chromosome in which the gene resided was homoeologous with the rice chromosome on which the rice orthologue resided, and (3) the bin location of the gene http://wheat.pw.usda.gov/cgi-bin/westsql/map_locus.cgi agreed with the location of the putative rice orthologue. If any ambiguity was encountered, the location of the gene on the *Brachypodium distachyon *and sorghum pseudomolecules [30,31] was taken into account. The loci inserted into the diversity maps on the basis of synteny with rice are indicated in Additional file 2, Table S1 by having no cM value assigned.

### Comparisons of genetic maps with wheat deletion-bin maps

The positions of genes on the *Ae. tauschii *genetic map were compared with their locations on the wheat deletion-bin maps compiled in the GrainGenes database http://wheat.pw.usda.gov/cgi-bin/westsql/map_locus.cgi. The deletion bins in Additional file 2, Table S1 were named according to the proximal breakpoint delimiting a bin. The proximal-most bin delimited by the centromeric break on the proximal side received the letter c (for centromeric) following the chromosome arm name. Bins within a chromosome were arbitrarily colored in Additional file 2, Table S1. If a gene was previously mapped into a wheat bin located in a different homoeologous group, the cell of the bin map was not colored. If no bin information was available for a locus, the cells were left blank in Additional file 2, Table S1. The bin location of a locus was considered inconsistent with the location of the locus on the deletion-bin map if it conflicted with the linear order of recombination blocks on the genetic map.

### Diversity estimation

Diversity was estimated only for mapped loci. The edited alignments of the 13 *T. aestivum *lines were compared. Nucleotide polymorphism *θ_w _*[98], nucleotide diversity *θ_π _*[18], average number of haplotypes per locus (*H*), haplotype diversity (*h*) [99], Tajima's *D *[100], and Wall's *B *[101] were computed for each gene using tools from the libsequence library [102]. The same descriptive statistics were computed for 10 lines representative of wild emmer in the Diyarbakir region and 9 accessions of synthetic wheat.

An unbiased estimation of diversity of a population requires the alignment of homologous sequences. This prerequisite is complicated in polyploid populations by the potential for inadvertently incorporating homoeologous sequences into the alignments, which would upwardly bias the estimates of average sequence diversity, sometimes dramatically. Coalescent simulations were used to estimate diversity variance expected under neutral coalescent histories. Simulations were performed in ms [103] and results were summarized using the msstats tool from the libsequence library [102]. For the A and B genomes, simulations were based on estimates of diversity in wild emmer, with the generative value of *θ *based on mean *θ *for each chromosome and the average length of amplicons for the same chromosome. A total of 10,000 simulations per chromosome was performed. The 99^th ^percentile of *θ_π _*from the 10,000 simulations was taken as the upper bound of *θ_π _*expected for each chromosome. Loci in both wild emmer and *T. aestivum *for which empirical estimates of *θ_π _*exceeded the upper bound were excluded from estimation of sequence diversity. Simulations for the D genome were based on the mean chromosome-specific estimates of *θ_π _*for the D genome of synthetic wheats. Loci for which empirical estimates of *θ_π _*in synthetic wheats exceeded the upper bound for the chromosome were excluded from further analysis in both synthetic wheats and *T. aestivum*. Loci excluded from computations are reported in Additional file 2, Table S1 but are indicted by a yellow cell color. Data containing less than 75% of the lines were also excluded from the analyses. They are reported in Additional file, Table S1 but are indicted by a red cell color.

The polydNdS program from libsequence [102] was used to estimate polymorphism at replacement and silent codon positions. The outputs estimated diversity for the whole gene, exons only, introns plus flanking sequences (UTRs) only, and replacement and silent polymorphisms. Only codons that differed at one position or codons that differed at two positions where the sites could be unambiguously assigned as synonymous or nonsynonymous were used.

The frequency spectrum of the less frequent (minor) allele was estimated in the sample of 10 homozygous wild emmer lines (10 chromosomes) and 13 homozygous *T. aestivum *lines (13 chromosomes). The distribution of the minor allele frequency in a sample of size *n *is described by the folded spectrum [36,104], which estimates the frequency of SNP sites with a minor allele in the *i*^th ^chromosome of *n *investigated chromosomes (*i *ranges from 1 to ≤ n/2). The folded spectra were computed for silent and replacement codon positions for individual genomes of wild emmer and *T. aestivum*.

### Statistical tests

Significance of differences among genomes was tested with the GLM and LSD procedures (SAS), using mean *θ*, the mean number of haplotypes (*H*) and mean haplotype diversity (*h*) per chromosome as variables. Because *θ*, *H*, and *h *across loci are not normally distributed, the GLM procedure could not be used to evaluate the significance of differences in these variables among chromosomes. The significance of differences between chromosome means was tested by estimating a 99% confidence interval (CI) about the genome mean of *θ, H*, and *h *from the distribution of 1000 means of random samples drawn with replacement from the population of *θ, H*, and *h *of genes within a genome (A, B, D genomes) nested within a species (*T. aestivum *and wild emmer). Chromosome means outside of the 99% CI were declared significantly different from the genome mean.

## Authors' contributions

EDA, ODA, JAA, MTC, JDu, BSG, YQG, MCL, PEM, COQ, LET, MLW, and JDv designed research; EDA and JDv coordinated research activity among the laboratories and PEM coordinated communication among the laboratories. CT, MLW, and JDv isolated genomic DNAs. FMY and ODA designed CP primers, ARA, NB, EJC, KRD, JH, HH, NH, DT, DMT, CT, and WZ performed PCR amplification, purification of amplicons and their submission for sequencing, alignment of sequences and design of GSPs; ODA, DCD, and CCC sequenced PCR amplicons. EDA, ODA, DEM, GRL, FMY, and JDv designed the project database and JR and FMY input data into the database. EDA, PLM, MTC, FMY, and JDv analyzed data. JDv drafted the manuscript. All authors read and approved the final version of the manuscript.

## Supplementary Material

Additional file 1 **Table S1 summarizes estimates of nucleotide polymorphism *θ*_w _and nucleotide diversity *θ*_π _at the replacement (*N*) and silent (*S*) codon positions, and noncoding portions of genes and the ratios of diversity at the replacement and silent codon positions in genes in the individual chromosomes of the A and B genomes of *T. dicoccoides *population from the Diyarbarkir region in Turkey**. Table S2 summarizes estimates of nucleotide polymorphism *θ*_w _and nucleotide diversity *θ*_π _at the replacement (*N*) and silent (*S*) codon positions and in noncoding portions of genes and the ratios of diversity at the replacement and silent codon positions in the A, B, and D genomes of *T. aestivum*. Figure S1 is a neighbor joining unrooted tree of 476 *T. aestivum *accessions constructed from Nei's genetic distances computed from RFLP at 131 loci. The tree depicts genetic relationships among 13 *T. aestivum *lines used for resequencing and SNP discovery. Figures S2 and S3 show the numbers of haplotypes per gene along the A-genome and B-genome chromosomes, respectively, in *T. aestivum *and wild emmer (*T. dicoccoides*) in the Diyarbakir region in Turkey. Figure S4 shows the numbers of haplotypes per gene along the D-genome chromosomes in *T. aestivum*. Figure S5 is a neighbor joining unrooted tree of 55 wild emmer (*T. dicoccoides*) accessions from the Diyarbakir region in Turkey constructed from Nei's genetic distances computed from RFLP at 153 loci. The tree depicts genetic relationships among 10 wild emmer accessions used for resequencing and SNP discovery in wild emmer. Click here for file

Additional file 2 **Table S1 is an Xcel table summarizing locus diversity measures in the A, B, and D genomes of *T. aestivum*, the A and B genomes of Diyarbakir population of wild emmer, and the D genome of synthetic wheats**. Table S1 further shows synteny of the diversity map with the wheat deletion-bin maps and the rice 12 pseudomolecules. Click here for file
