# Supplementary material for: Nucleotide diversity maps reveal variation in diversity among wheat genomes and chromosomes
Source: BMC Genomics. 2010 Dec 14;11:702. doi: 10.1186/1471-2164-11-702 (PMC3022916; doi:10.1186/1471-2164-11-702)
Supplement: Additional file 1 — Table S1 summarizes estimates of nucleotide polymorphism θw and nucleotide diversity θπ at the replacement (N) and silent (S) codon positions, and noncoding portions of genes and the ratios of diversity at the replacement and silent codon positions in genes in the individual chromosomes of the A and B genomes of T. dicoccoides population from the Diyarbarkir region in Turkey. Table S2 summarizes estimates of nucleotide polymorphism θw and nucleotide diversity θπ at the replacement (N) and silent (S) codon positions and in noncoding portions of genes and the ratios of diversity at the replacement and silent codon positions in the A, B, and D genomes of T. aestivum. Figure S1 is a neighbor joining unrooted tree of 476 T. aestivum accessions constructed from Nei's genetic distances computed from RFLP at 131 loci. The tree depicts genetic relationships among 13 T. aestivum lines used for resequencing and SNP discovery. Figures S2 and S3 show the numbers of haplotypes per gene along the A-genome and B-genome chromosomes, respectively, in T. aestivum and wild emmer (T. dicoccoides) in the Diyarbakir region in Turkey. Figure S4 shows the numbers of haplotypes per gene along the D-genome chromosomes in T. aestivum. Figure S5 is a neighbor joining unrooted tree of 55 wild emmer (T. dicoccoides) accessions from the Diyarbakir region in Turkey constructed from Nei's genetic distances computed from RFLP at 153 loci. The tree depicts genetic relationships among 10 wild emmer accessions used for resequencing and SNP discovery in wild emmer. [file 1471-2164-11-702-S1.DOC]

**Additional file 1**

Table S1: Nucleotide polymorphism *θ*_w_ and nucleotide diversity *θ*_π_ at the replacement (*N*) and silent (*S*) codon positions, noncoding portions of genes, and the ratios of diversity at the replacement and silent codon positions in *n* number of genes in individual chromosomes of the A and B genomes of *T. dicoccoides* population from the Diyarbarkir region in Turkey.

|  |  | Coding (× 10^-3^) | | Noncoding (× 10^-3^) | | *N* (× 10^-3^) | | *S* (× 10^-3^) | | *N/S* | |
| --- | --- | --- | --- | --- | --- | --- | --- | --- | --- | --- | --- |
| Chrom. | *n* | *θ_w_* | *θ_π_* | *θ_w_* | *θ_π_* | *θ_w_* | *θ_π_* | *θ_w_* | *θ_π_* | *θ_w_* | *θ_π_* |
| 1A | 43 | 0.71 | 0.71 | 1.10 | 1.16 | 0.30 | 0.41 | 2.21 | 1.78 | 0.13 | 0.23 |
| 2A | 58 | 0.63 | 0.65 | 1.57 | 1.97 | 0.32 | 0.35 | 1.17 | 1.67 | 0.19 | 0.21 |
| 3A | 38 | 0.57 | 0.62 | 0.62 | 0.64 | 0.44 | 0.48 | 1.03 | 1.15 | 0.43 | 0.42 |
| 4A | 90 | 0.32 | 0.30 | 0.68 | 0.55 | 0.16 | 0.15 | 0.91 | 0.86 | 0.17 | 0.17 |
| 5A | 52 | 0.60 | 0.58 | 0.83 | 0.82 | 0.27 | 0.24 | 1.85 | 1.86 | 0.15 | 0.13 |
| 6A | 44 | 0.56 | 0.55 | 0.57 | 0.63 | 0.38 | 0.43 | 1.14 | 0.96 | 0.33 | 0.44 |
| 7A | 65 | 0.67 | 0.78 | 1.01 | 1.10 | 0.40 | 0.44 | 1.67 | 2.03 | 0.24 | 0.21 |
| 1B | 45 | 0.60 | 0.64 | 2.33 | 2.41 | 0.47 | 0.50 | 1.14 | 1.16 | 0.41 | 0.43 |
| 2B | 44 | 0.73 | 0.89 | 1.21 | 1.17 | 0.36 | 0.38 | 1.96 | 2.62 | 0.18 | 0.15 |
| 3B | 39 | 0.47 | 0.45 | 1.24 | 1.12 | 0.17 | 0.25 | 1.77 | 1.28 | 0.10 | 0.20 |
| 4B | 92 | 0.50 | 0.39 | 0.79 | 0.67 | 0.50 | 0.38 | 0.53 | 0.43 | 0.94 | 0.88 |
| 5B | 43 | 0.52 | 0.45 | 0.66 | 0.62 | 0.24 | 0.24 | 1.48 | 1.18 | 0.16 | 0.20 |
| 6B | 33 | 0.51 | 0.50 | 0.95 | 0.90 | 0.15 | 0.17 | 1.74 | 1.66 | 0.09 | 0.10 |
| 7B | 57 | 0.60 | 0.57 | 1.29 | 1.29 | 0.27 | 0.17 | 1.69 | 1.88 | 0.16 | 0.09 |

Table S2: Nucleotide polymorphism *θ*_w_ and nucleotide diversity *θ*_π_ at the replacement (*N*) and silent (*S*) codon positions and in noncoding portions of genes and the ratios of diversity at the replacement and silent codon positions in the *A*, *B*, and *D* genomes of *T. aestivum*

|  |  | Coding | | Noncoding | | *N* | | *S* | | *N*/*S* | |
| --- | --- | --- | --- | --- | --- | --- | --- | --- | --- | --- | --- |
| Chrom. | *n* | *θ_w_* | *θ_π_* | *θ_w_* | *θ_π_* | *θ_w_* | *θ_π_* | *θ_w_* | *θ_π_* | *θ_w_* | *θ_π_* |
| 1A | 57 | 0.56 | 0.67 | 1.06 | 0.93 | 0.30 | 0.34 | 1.54 | 1.91 | 0.19 | 0.18 |
| 2A | 39 | 0.41 | 0.45 | 0.83 | 0.80 | 0.26 | 0.25 | 0.99 | 1.28 | 0.27 | 0.19 |
| 3A | 89 | 0.74 | 0.77 | 0.59 | 0.53 | 0.46 | 0.58 | 1.55 | 1.27 | 0.30 | 0.46 |
| 4A | 49 | 0.32 | 0.35 | 0.53 | 0.49 | 0.23 | 0.24 | 0.66 | 0.76 | 0.35 | 0.32 |
| 5A | 49 | 0.67 | 0.69 | 1.08 | 1.10 | 0.17 | 0.11 | 2.49 | 2.77 | 0.07 | 0.04 |
| 6A | 45 | 0.63 | 0.60 | 0.51 | 0.58 | 0.40 | 0.36 | 1.34 | 1.41 | 0.30 | 0.25 |
| 7A | 62 | 0.48 | 0.32 | 0.75 | 0.58 | 0.31 | 0.19 | 1.12 | 0.79 | 0.28 | 0.24 |
| 1B | 44 | 0.88 | 1.08 | 1.06 | 1.18 | 0.70 | 0.84 | 1.60 | 2.04 | 0.44 | 0.41 |
| 2B | 44 | 0.94 | 1.17 | 1.61 | 1.66 | 0.51 | 0.66 | 2.47 | 2.97 | 0.21 | 0.22 |
| 3B | 39 | 0.60 | 0.68 | 0.93 | 0.95 | 0.35 | 0.53 | 1.72 | 1.42 | 0.20 | 0.37 |
| 4B | 94 | 0.18 | 0.09 | 0.23 | 0.17 | 0.09 | 0.04 | 0.49 | 0.27 | 0.17 | 0.15 |
| 5B | 40 | 0.50 | 0.48 | 0.49 | 0.49 | 0.19 | 0.19 | 1.56 | 1.48 | 0.12 | 0.12 |
| 6B | 34 | 0.43 | 0.32 | 0.94 | 0.97 | 0.21 | 0.12 | 1.23 | 1.05 | 0.17 | 0.12 |
| 7B | 56 | 0.30 | 0.29 | 0.94 | 0.83 | 0.17 | 0.13 | 0.74 | 0.82 | 0.23 | 0.16 |
| 1D | 54 | 0.28 | 0.27 | 0.60 | 0.60 | 0.13 | 0.14 | 0.80 | 0.75 | 0.17 | 0.18 |
| 2D | 55 | 0.50 | 0.42 | 0.60 | 0.47 | 0.12 | 0.13 | 1.85 | 1.45 | 0.07 | 0.09 |
| 3D | 51 | 0.41 | 0.23 | 0.23 | 0.18 | 0.05 | 0.03 | 1.72 | 0.98 | 0.03 | 0.03 |
| 4D | 84 | 0.07 | 0.06 | 0.25 | 0.18 | 0.02 | 0.01 | 0.24 | 0.25 | 0.08 | 0.04 |
| 5D | 53 | 0.01 | 0.01 | 0.20 | 0.17 | 0.00 | 0.00 | 0.06 | 0.03 | 0.00 | 0.00 |
| 6D | 44 | 0.08 | 0.09 | 0.41 | 0.48 | 0.05 | 0.06 | 0.18 | 0.21 | 0.26 | 0.26 |
| 7D | 62 | 0.22 | 0.16 | 0.25 | 0.15 | 0.08 | 0.08 | 0.70 | 0.46 | 0.12 | 0.17 |


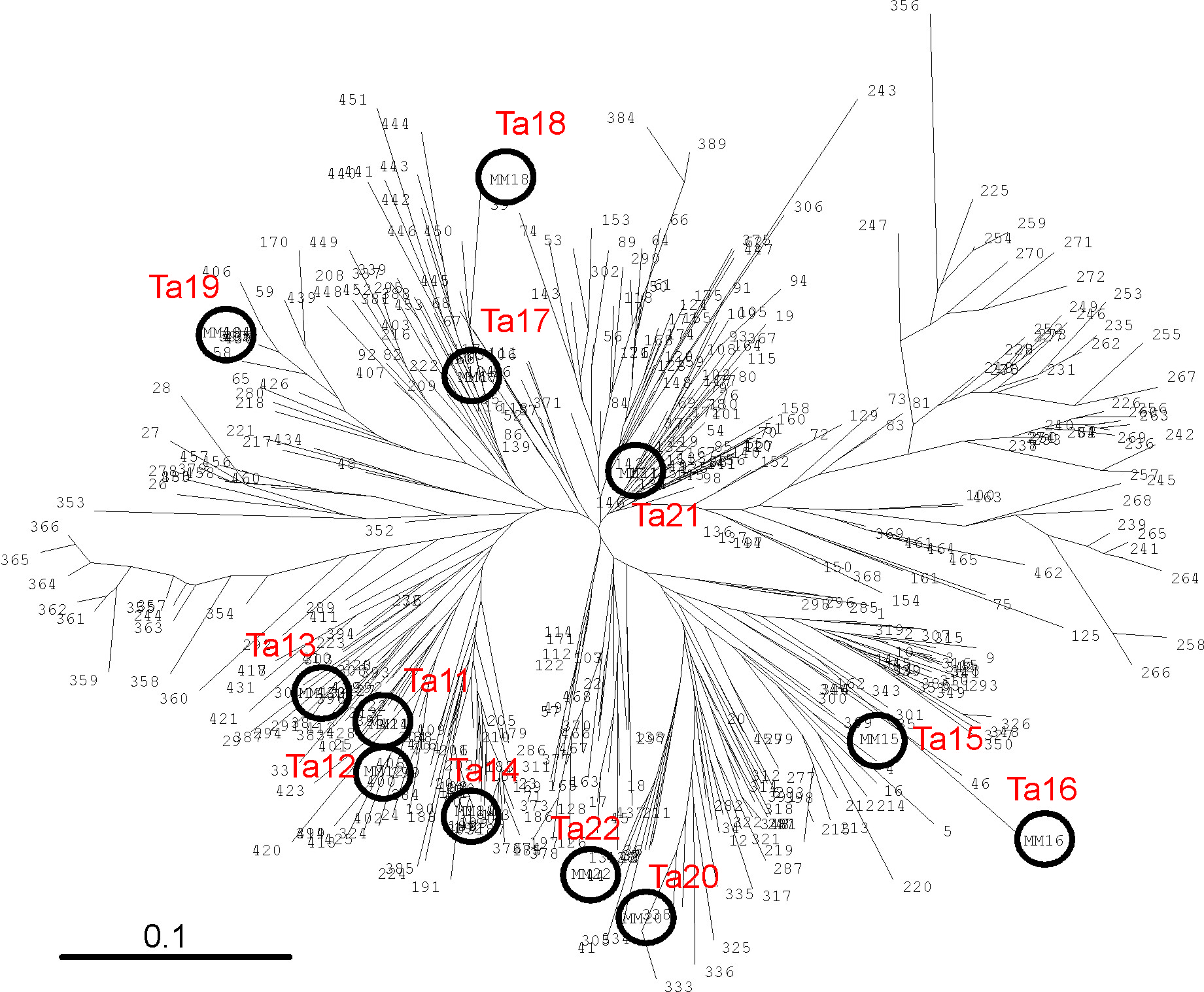


Figure S1: A neighbor joining unrooted tree of 476 *T. aestivum* accessions constructed from Nei’s genetic distances computed from RFLP at 131 loci. The locations of accessions selected for SNP discovery are indicated by circles and code names, which correspond to those in Table 10. Note the central location of Chinese Spring (Ta21). The horizontal bar indicates a genetic distance of 0.1. Only 12 lines are shown because Opata was not included into the study.


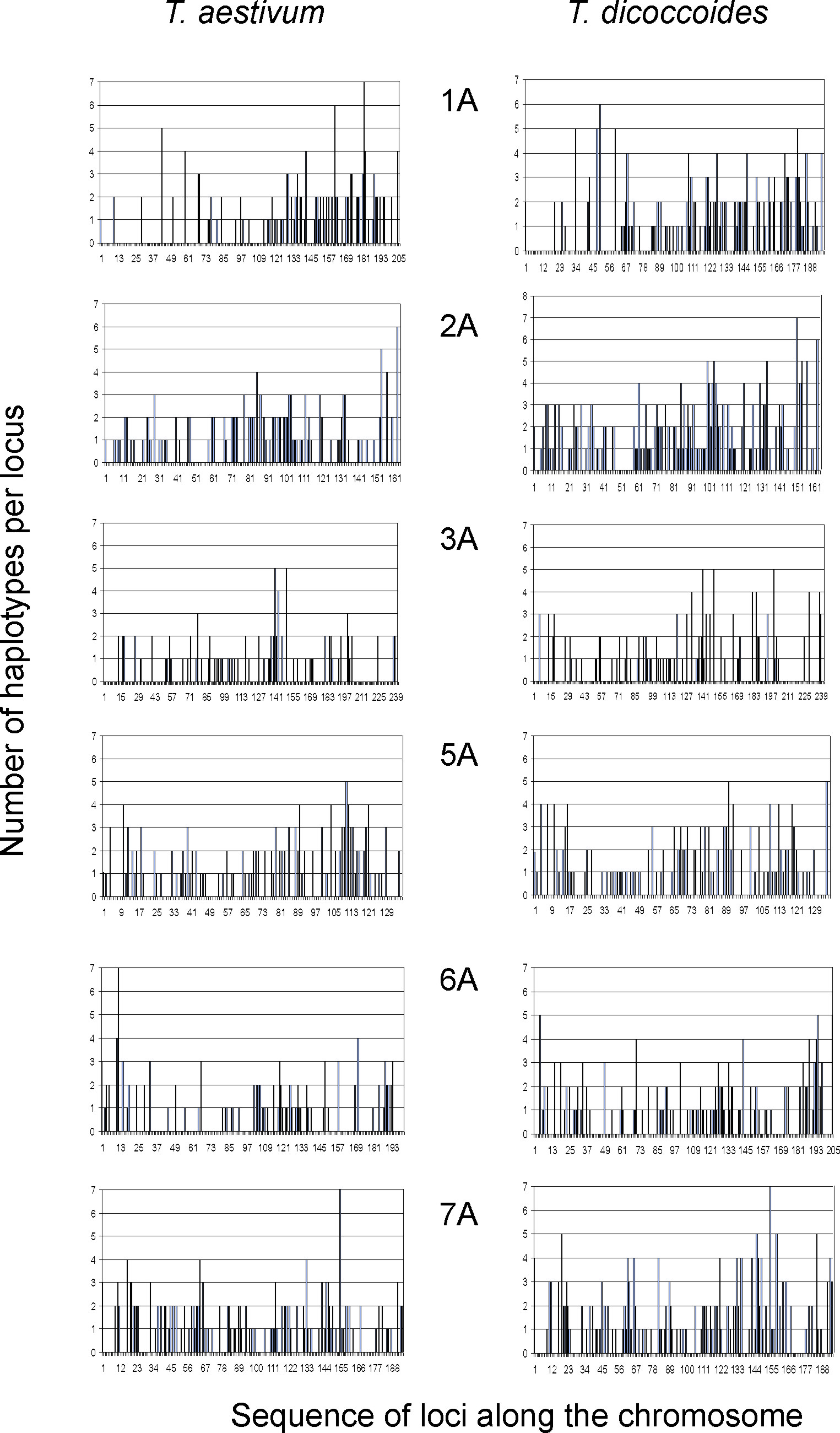


Figure S2: The numbers of haplotypes per gene along the A-genome chromosomes in *T. aestivum* and wild emmer (*T. dicoccoides*) in the Diyarbakir region in Turkey. Chromosome 4A is excluded because the order of genes does not conform to the *Ae. tauschii* genetic map. The gene order along the diversity maps in Additional file 2, Table 1 is used on the X axis, and the maps are oriented with the most distal gene in the short arm to the left. Genetic distances between genes are disregarded but all loci on the diversity maps are included. Loci for which no diversity estimate exists in Additional file 1 show zero number of haplotypes


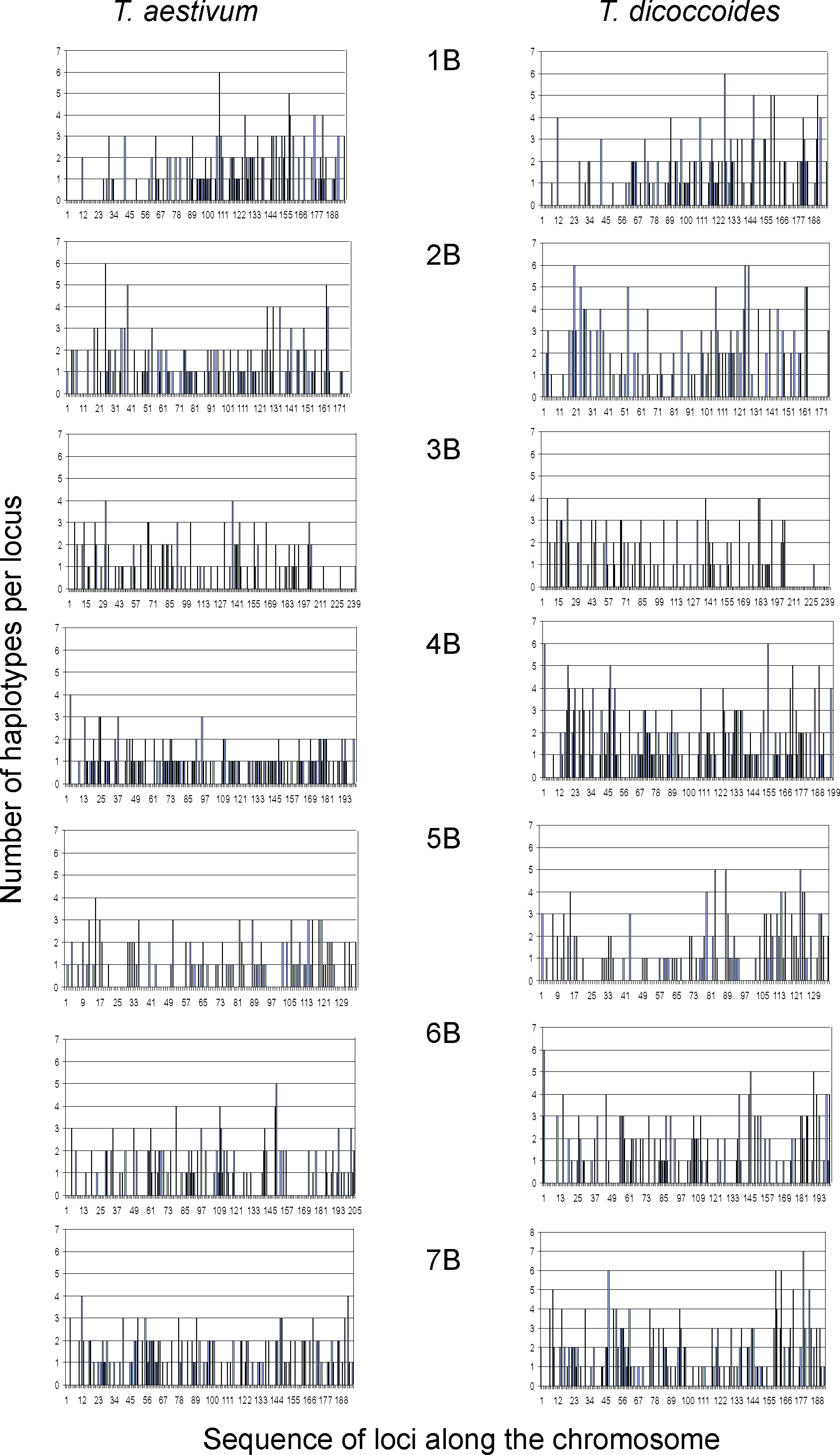


Figure S3: The numbers of haplotypes per gene along the B-genome chromosomes in *T. aestivum* and wild emmer (*T. dicoccoides*) in the Diyarbakir region in Turkey. See Additional file 1, Figure S2 for details.


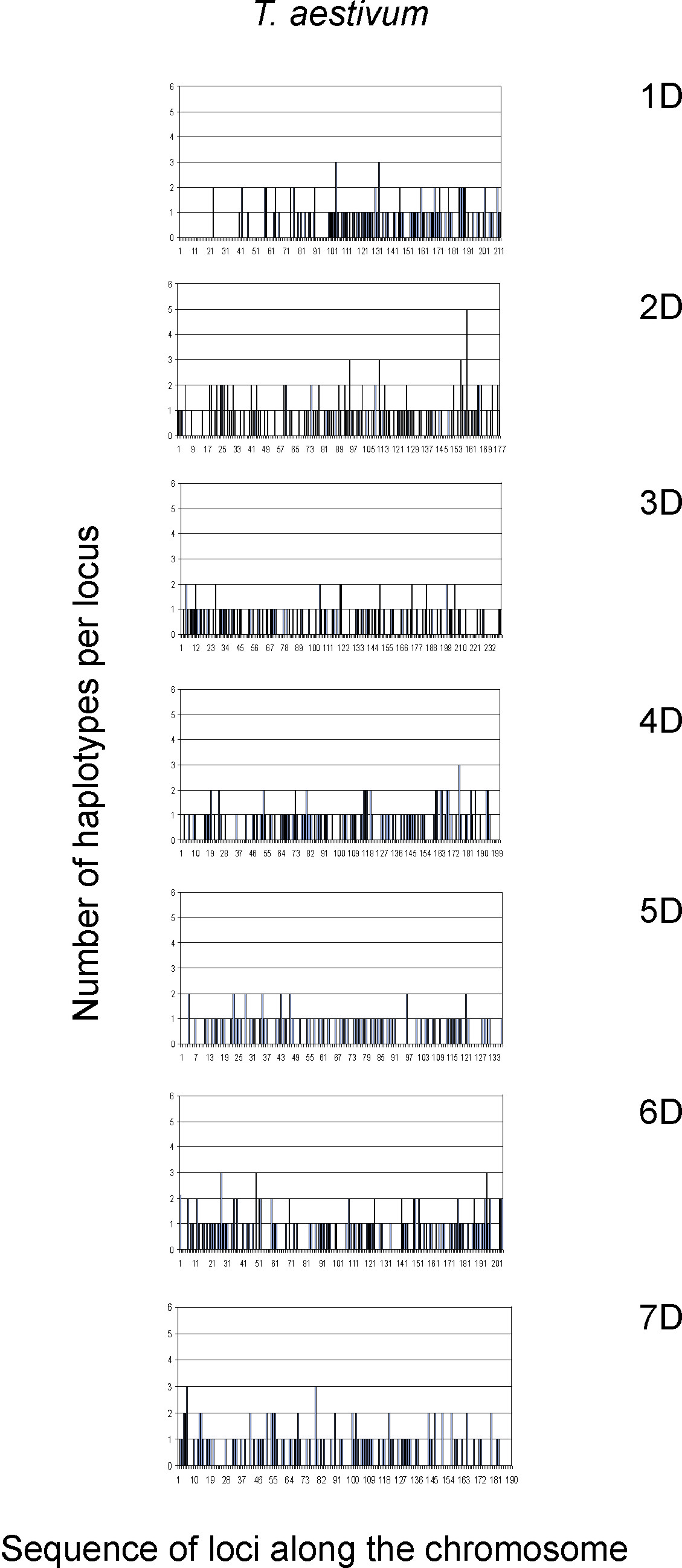


Figure S4: The numbers of haplotypes per gene along the *D*-genome chromosomes in *T. aestivum.* See Additional file 1, Figure S2 for details.


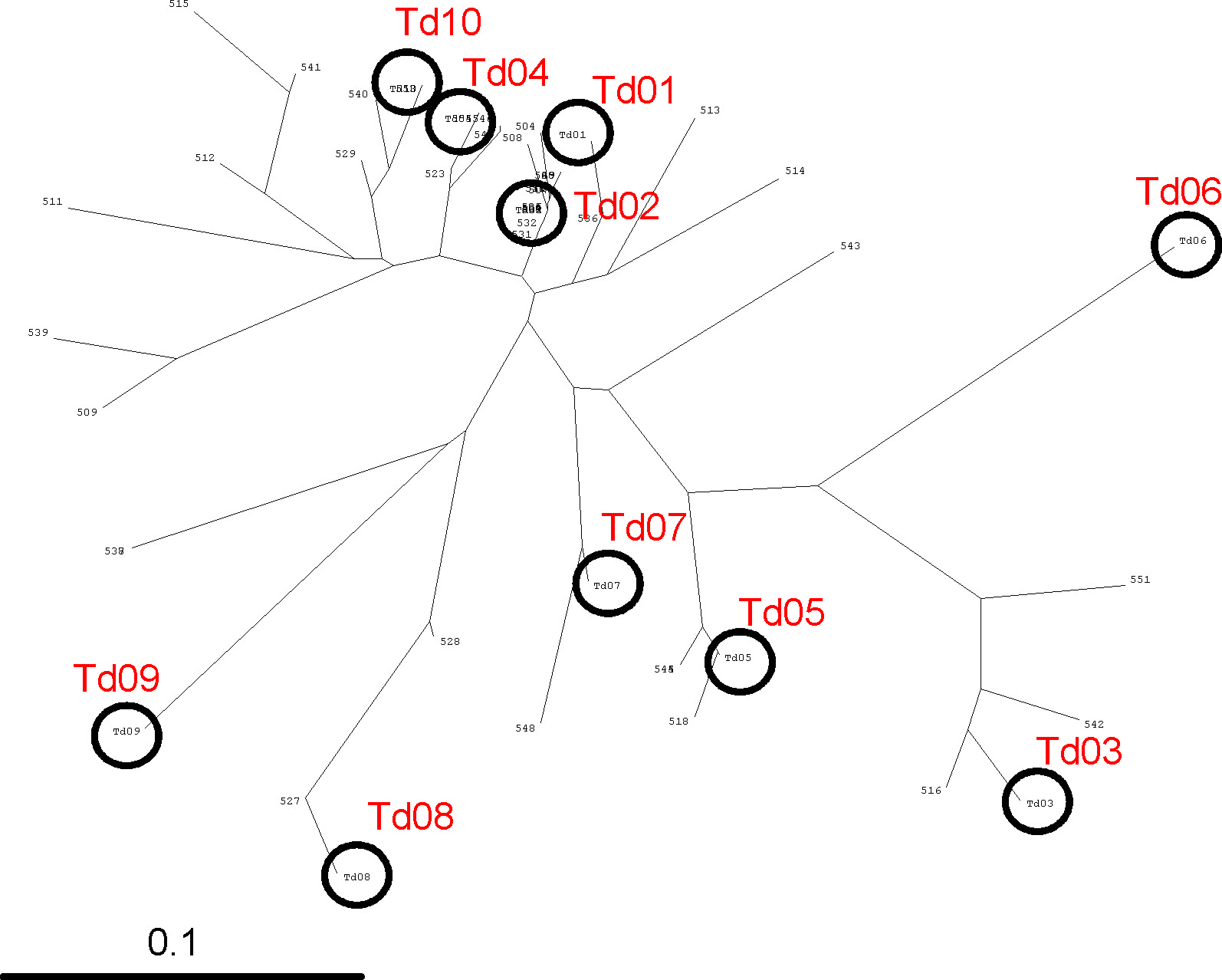


Figure S5: A neighbor joining unrooted tree of 55 wild emmer (*T. dicoccoides*) accessions from the Diyarbakir region in Turkey constructed from Nei’s genetic distances computed from RFLP at 153 loci. The locations of accessions selected for SNP discovery are indicated by circles. Code names correspond to those in Table 10. The horizontal bar indicates a genetic distance of 0.1.
